# Sources, distribution and fate of microfibres on the Great Barrier Reef, Australia

**DOI:** 10.1038/s41598-019-45340-7

**Published:** 2019-06-21

**Authors:** Lene H. Jensen, Cherie A. Motti, Anders L. Garm, Hemerson Tonin, Frederieke J. Kroon

**Affiliations:** 1 Australian Institute of Marine Science, Townsville, Qld 4810 Australia; 2 University of Copenhagen, Universitetsparken 4, 2100 Copenhagen, Denmark

**Keywords:** Tropical ecology, Environmental impact, Marine biology, Characterization and analytical techniques

## Abstract

Marine microdebris, in particular microplastics (plastics <5 mm), has become an issue of international concern due to its prevalence, persistence and potential adverse impacts on marine ecosystems. Informing source reduction based on ecological effects requires an understanding of the origin, distribution and characteristics of microdebris and the interactions with marine organisms. Here we show widespread contamination of the central Great Barrier Reef environment with microdebris, with microfibres comprising 86% of all items detected. Microdebris intake by coral reef fish was non-random, with chemical composition, shape and colour differing significantly from that detected in surface waters. Furthermore, the origin of microdebris contamination in surface waters is non-random with riverine discharge a likely source for microdebris detected at inshore, but not at offshore reef locations. Our findings demonstrate the complexities associated with determining marine microdebris exposure and fate, and assist in improving future ecological assessments and prioritizing source reduction.

## Introduction

Contamination of marine and coastal environments with marine debris is pervasive and has been documented in habitats, organisms and ecosystems around the world^1^. Marine debris can consist of many different materials that have been manufactured, modified or used by humans, however, plastic items are generally the most common type^2,3^. Recently the presence of microplastics (i.e. plastics <5 mm) has received increasing attention and is considered an emerging issue of international concern^4^. Microplastic contamination of the marine environment has been reported globally, including for coastlines^5^, sea surface and water column^6,7^, and benthic sediments^2^. Its pervasiveness raises concerns about the potential adverse impacts on marine organisms and ecosystems^8^, with intake of microplastics documented in a large number of wild caught organisms^9,10^. Few field studies, however, have examined interactions between microplastic exposure and intake by marine organisms^11^.

The main entry points of microplastics into the marine environment are rivers, coastlines, marine and atmospheric sources^4^. At a global scale estimates of plastic input have been reported for rivers^12^ and coastlines^13^, but their contribution to marine microplastic contamination, including relative to other sources, remains unclear^14,15^. The magnitude of ocean contamination with microplastics is emphasised by recent estimates, ranging from 5 to 51 × 10^12^ particles and weighing between 36 and 236 × 10^3^ tonnes^6,16^. Results from global ocean transport models and observational data indicate that this debris preferentially accumulates in the five convergence zones in the subtropical latitudes of the Pacific, Atlantic and Indian Ocean basins^17,18^. At regional and local scales, numerical simulations to determine potential sources, distribution and fate of microplastics also reveal clear pathways and accumulation areas^19–21^. While such exercises are sometimes combined with microplastic monitoring efforts^19,20^, we are not aware of any numerical simulation studies that have examined both the potential sources, and the spatial and temporal distribution of microplastics and its co-occurrence with marine organisms.

To relate microplastic intake by marine organisms with microplastic exposure in the marine environment, detailed examination of their chemical and physical characteristics is required^22,23^. The main chemical characteristic of microplastics to confirm plastic origin, and infer potential source material, is polymer type and additives^22,23^. Infrared spectroscopy has been recommended as the most reliable method to identify the chemical composition of microplastics^22^, and will assist in eliminating false positives in environmental samples^24,25^. Polyethylene (PE), polypropylene (PP) and polystyrene (PS) appear to be the most common polymer type of microplastics detected in marine ecosystems^22^, reflecting global plastic production and usage in packaging and fishing materials^26,27^. Nevertheless, spectroscopy is often not used to confirm the polymer type of putative microplastics^28^, and as a result the relative abundance of synthetic polymers in marine microdebris has recently been called into question^29^. The main physical characteristics of microplastics reported in the literature include size, shape and colour^23^, with stereo-microscopy commonly used to describe and quantify these features^22^. Plastic cylindrical pellets were the first reported microplastics in surface waters^30^, however, more recent work has demonstrated that fibres appear to be the most common shape in marine microdebris^15^. Spectroscopy often confirms that the majority of fibres detected in marine surface waters, sediment and organisms are not all synthetic plastics but also contain semi-synthetic items such as rayon^29,31,32^. In addition to informing preferential intake and potential fate of microplastics in marine ecosystems, detailed examination of their chemical and physical characteristics will also elucidate potential source materials.

**Figure 1:**
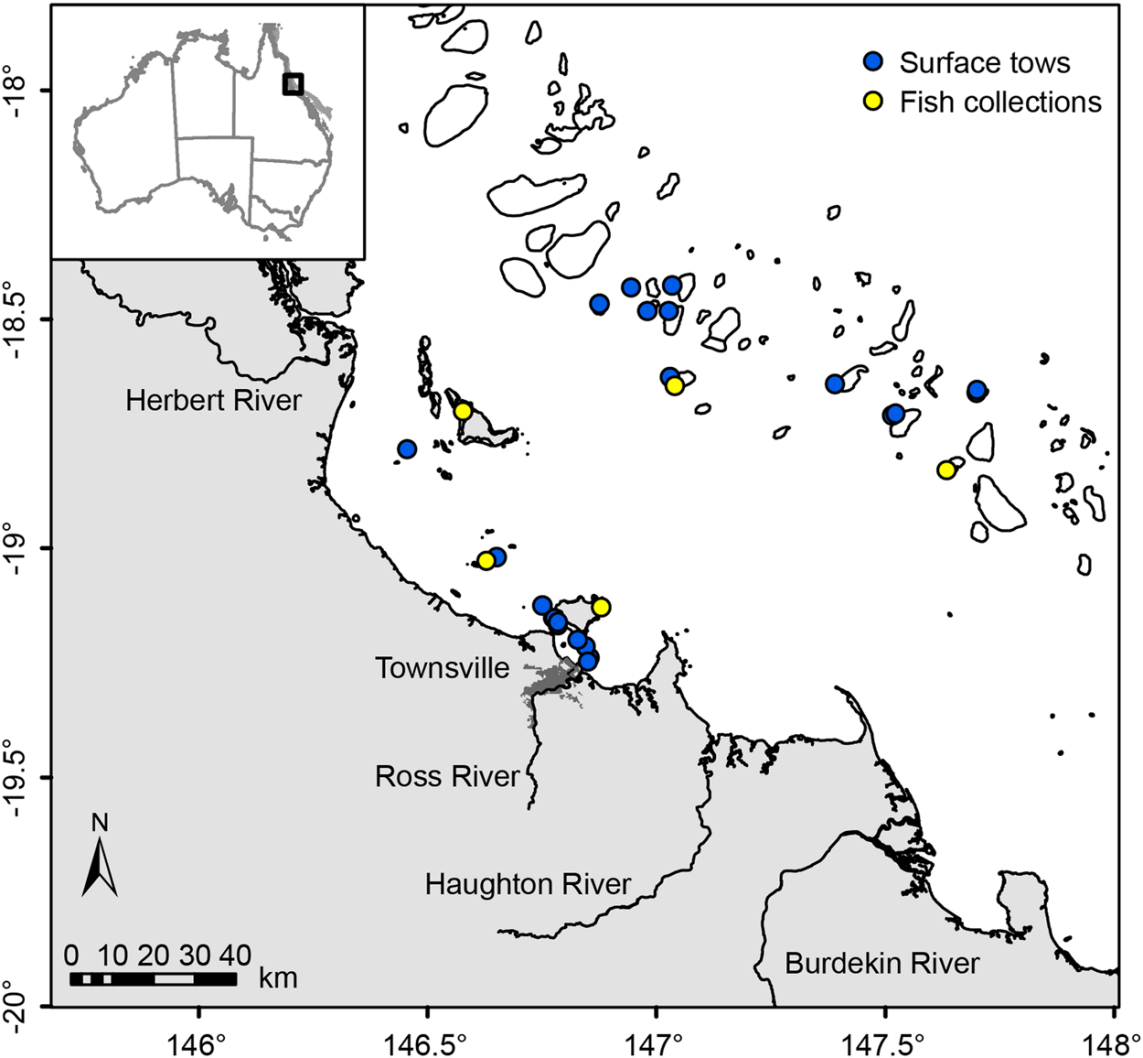
Sampling locations. Locations of surface water tows and Lemon damselfish collections at inshore and offshore reefs of the central Great Barrier Reef World Heritage Area, Australia, conducted in 2016. Insert shows location of study area in Australia.

Here, we relate intake of marine microdebris by a common, small coral reef fish with microdebris exposure in surface waters of the central Great Barrier Reef (GBR) World Heritage Area (WHA), Australia. We collected 22 surface water samples and 60 Lemon damselfish (*Pomacentrus moluccensis*) at several inshore and offshore reef locations geographically separated by the GBR lagoon (Fig. 1; Table S1). Lemon damselfish was chosen as our study species, as it is site-attached with a small home range of a few square meters^33^ suggesting that any intake of marine microdebris reflects local exposure. To characterise marine microdebris contamination in these samples, we applied a consistent analysis workflow developed for estimating and classifying marine microdebris contamination^25,29^. Specifically, potential marine microdebris items in surface water and Lemon damselfish were visually separated from their surrounding sample matrix using stereomicroscopy, physically characterised using microscopic photography, and chemically characterised using attenuated total reflectance (ATR) Fourier transform infrared (FTIR) spectroscopy^25,29^. Furthermore, we conducted two numerical simulations with a hydrodynamic model to examine the potential role of riverine discharges as likely sources, and the potential transport pathways and fate of floating marine microdebris detected at the 22 surface water sampling locations. Overall, the aim of the study is to demonstrate the diversity in origin, characteristics and distribution of marine microdebris present in the central GBR environment, and to elucidate the need for improved ecological assessments to inform source reduction.

## Results

### Chemical and physical characterisation of marine microdebris

**Figure 2:**
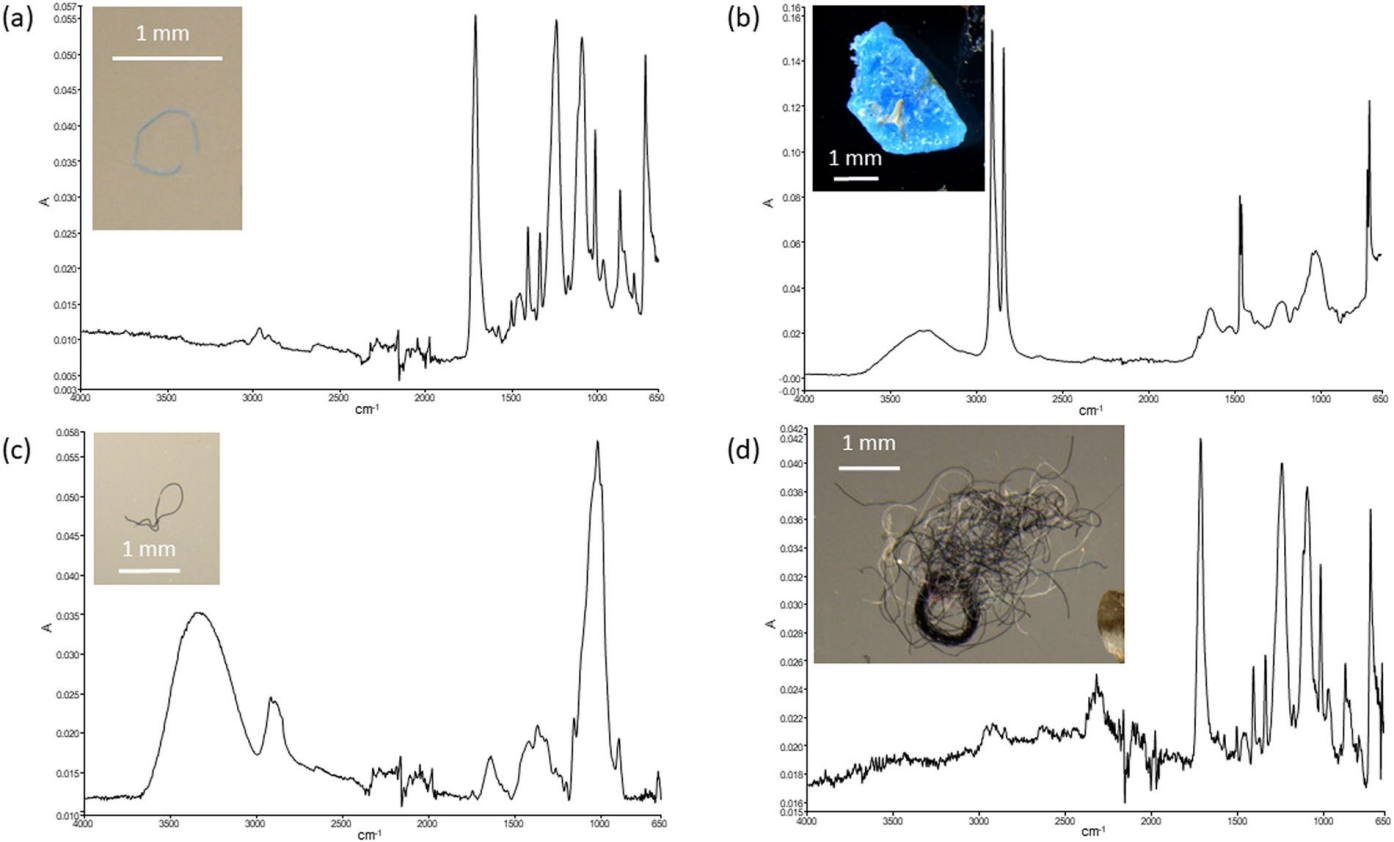
ATR-FTIR spectral matches of representative marine microdebris particles and fibres. Attenuated total reflectance (ATR) Fourier transform infrared (FTIR) spectral matches and photographs for representative marine microdebris particles and fibres detected in surface water tows (a + b) and Lemon damselfish, *Pomacentrus moluccensi*s (c + d), namely (**a**) blue polyester fibre from Ross River estuary (inshore), (**b**) blue polyethylene particle from Centipede Reef (offshore), (**c**) black rayon fibre from Magnetic Island (inshore), and (**d**) nest of fibres containing polyester from John Brewer Reef (offshore). Samples were collected at coastal inshore and offshore reef locations in the central region of the Great Barrier Reef World Heritage Area, Australia, 2016.

In the 22 surface water tows conducted near eleven inshore and eleven offshore reefs a total of 547 marine microdebris items were detected. This comprised 80% of the 681 potential marine microdebris items first separated visually from the filtered seawater samples using stereomicroscopy (see Supplementary Text, Tables S2–S4), emphasising the importance of using infrared spectroscopy to establish their chemical nature^24,25^. The 547 marine microdebris items consisted of a combination of synthetic (n = 195, 36%), semi-synthetic (n = 215, 39%), and naturally-derived (n = 137, 25%) chemical types. More than half of the items (n = 327, 60%) contained synthetic (i.e. plastic) polymers, including 132 natural fibre reinforced polymer composites (NFPC^34^), the most common being polyester (n = 86; Fig. 2a), nylon (n = 79) and PE (n = 52; Fig. 2b) (Table 1). Fibres (n = 411, 75%) were overall much more abundant than particles (n = 136, 25%), in particular among semi-synthetic and naturally-derived microdebris. The most abundant chemical types among semi-synthetic fibres were NFPCs, particularly those containing nylon (n = 79), and cellulose-regenerated single and composite fibres (i.e. rayon) without synthetics (n = 69). Among naturally-derived fibres, cellulose was by far the most common chemical type both as single and composite fibres (n = 103). In contrast, among synthetic microdebris PE particles (n = 51) were most common followed by polyester fibres (n = 36).

**Table 1:** Chemical type assignment of marine microdebris in Great Barrier Reef surface waters and Lemon damselfish.

Chemical type	Surface tows			Lemon damselfish		
	Fibres	Particles	Total	Fibres	Particles	Total
***SYNTHETIC***						
*Thermoplastics* ^§^						
Nylon	1	0	1	2	0	2
Plastic precursor	2	0	2	0	0	0
Polyethylene (PE)*	1	51	52	3	0	3
Polyethylene terephthalate (PET)	10	0	10	21	0	21
Polyethylene vinyl acetate (PEVA)*	0	12	12	0	0	0
Polypropylene	16	11	27	0	0	0
Polypropylene:acrylonitrile	0	0	0	0	0	0
Polypropylene:acrylonitrile:polyester	0	1	1	0	0	0
Poly(vinyl acetate) (PVA)*	0	2	2	1	0	1
Polyvinyl chloride (PVC)	1	6	7	0	0	0
*Thermoset and elastomer plastics* ^§^						
Acrylic paint	0	7	7	0	0	0
Acrylonitrile	2	0	2	1	0	1
Elastan	0	0	0	1	0	1
Epoxy	0	7	7	0	0	0
Magnesium stearate*	0	1	1	0	0	0
Plasticiser	0	0	0	1	0	1
Polybutadiene rubber*	1	7	8	0	0	0
Polyester	36	1	37	18	0	18
Polyester:elastan	5	0	5	4	0	4
Polyester:polyurethane	0	1	1	0	0	0
Polyurethane (PU)*	0	12	12	0	0	0
Resin	0	0	0	0	0	0
Soft co-polymer	0	1	1	3	0	3
*Total synthetic*	75	120	195	55	0	55
***SEMI-SYNTHETIC***						
*Regenerated*						
Cellulose-regenerated (rayon)	34	10	44	22	0	22
*Regenerated composites*						
Cellulose-regenerated (rayon):cellulose	31	0	31	60	0	60
Cellulose-regenerated (rayon):keratin	4	0	4	7	1	8
*NFPCs*						
NFPC:asphalt^#^	3	1	4	3	0	3
NFPC:acrylonitrile	7	0	7	10	0	10
NFPC:elastan	6	0	6	19	0	19
NFPC:nylon	26	0	26	8	0	8
NFPC:nylon:acrylonitrile	17	0	17	1	0	1
NFPC:nylon:elastan	33	0	33	3	0	3
NFPC:nylon:polyester	3	0	3	2	0	2
NFPC:polyester	27	2	29	5	0	5
NFPC:polyester:elastan	9	0	9	10	0	10
NFPC:polypropylene:polyester	2	0	2	2	0	2
*Total semi-synthetic*	202	13	215	152	1	153
***NATURALLY-DERIVED***						
*Plants*						
Cellulose	71	0	71	135	0	135
Dye	1	0	1	0	0	0
*Animals*						
Keratin	17	1	18	5	0	5
*Composites*						
Cellulose:cellulose	32	0	32	102	0	102
Cellulose:keratin	13	0	13	5	0	5
*Inorganics*						
Silicate	0	2	2	0	0	0
*Total naturally-derived*	134	3	137	247	0	247
Total	411	136	547	454	1	455

^§^main plastic property but can be interchangeable; *plastics less dense than seawater; ^#^does not contain any synthetic (i.e. plastic) polymers.

Presence of marine microdebris was examined in 22 surface water tows and in the gastrointestinal tract of 60 Lemon damselfish, *Pomacentrus moluccensis*, collected near inshore and offshore reefs in the central region of the Great Barrier Reef World Heritage Area, Australia, in 2016. Assignment was based on the chemical type as determined by ATR-FTIR, results from compare analyses, and visual inspection of photographs. Items were classified as synthetic, semi-synthetic, or naturally-derived. NFPC = natural fibre reinforced polymer composites.

In the gastrointestinal tract (GIT) of the 60 Lemon damselfish collected at three inshore reefs (n = 30) and two offshore reefs (n = 30), a total of 455 marine microdebris items were detected. Similar to surface waters, this comprised 82% of the 556 items of potential marine microdebris items first separated visually from the GIT content using stereomicroscopy (see Supplementary Text, Table S2–S4). All but one out of the 455 microdebris items detected in fish GITs were fibres (Table 1), and their observed frequency was significantly higher than those in surface waters (*Chi*-square = 138.5, df = 2, P < 0.0000001). Furthermore, only a quarter (n = 115, 25%) of all 455 microdebris items contained synthetic (i.e. plastic) polymers, including 60 NFPC fibres; polyester (n = 41) was by far the most common plastic (Table 1). The observed frequencies of synthetic, semi-synthetic and naturally-derived items in fish GIT differed significantly from those detected in surface waters (*Chi*-square = 206, df = 2, P < 0.0000001), with synthetic items less frequently and naturally-derived items more frequently observed. Among naturally-derived and semi-synthetic items, single and composite cellulose fibres (n = 237), and single and composite rayon fibres without synthetics (n = 89), respectively, were most common (Fig. 2c). Among synthetic fibres, polyester (n = 22; Fig. 2d) and polyethylene terephthalate (PET) (n = 21) were the most abundant plastic polymer type.

Based on their physical characteristics such as shape and colour^22^, all particles containing plastic polymers detected in surface waters and in damselfish GITs appeared to be fragments resulting from the breakdown of larger items. Primary microplastics such as microbeads from personal care products or pre-production plastic pellets were not observed.

### Presence and abundance of marine microdebris

**Figure 3:**
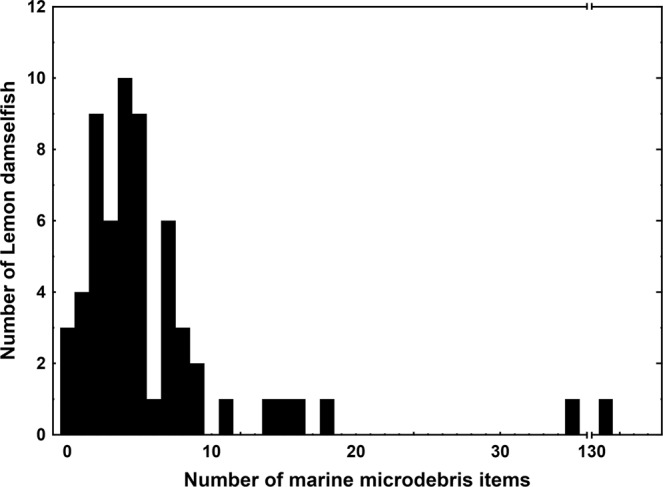
Frequency of individual Lemon damselfish with number of ingested marine microdebris items. Marine microdebris items were detected in the gastrointestinal tracts in 57 out of the 60 individual Lemon damselfish, *Pomacentrus moluccensis*, collected at five inshore and offshore reef locations in the central region of the Great Barrier Reef World Heritage Area, Australia, 2016.

Marine microdebris was detected in all 22 surface tows, and in 57 of the 60 Lemon damselfish examined. Surface waters contained an average of 24.9 ± 3.3 standard error (s.e.m.) items per tow (range: 5–57), with estimated concentration of marine microdebris items (*C*_*s*_) per tow ranging from 0.04 to 0.48 m^−3^ (Table S5a). Lemon damselfish GITs most often contained four items, ranging from 0 to 131 items (Fig. 3, Table S5b), many of which were entangled with other gut content. In one fish, a nest of fibres was observed (Fig. 2d) which was carefully separated to a total of 131 fibres. The total number of microdebris items detected in individual fish did not change with fish size (Spearman Rank Correlation, r_s_ = −0.12, P > 0.05).

**Figure 4:**
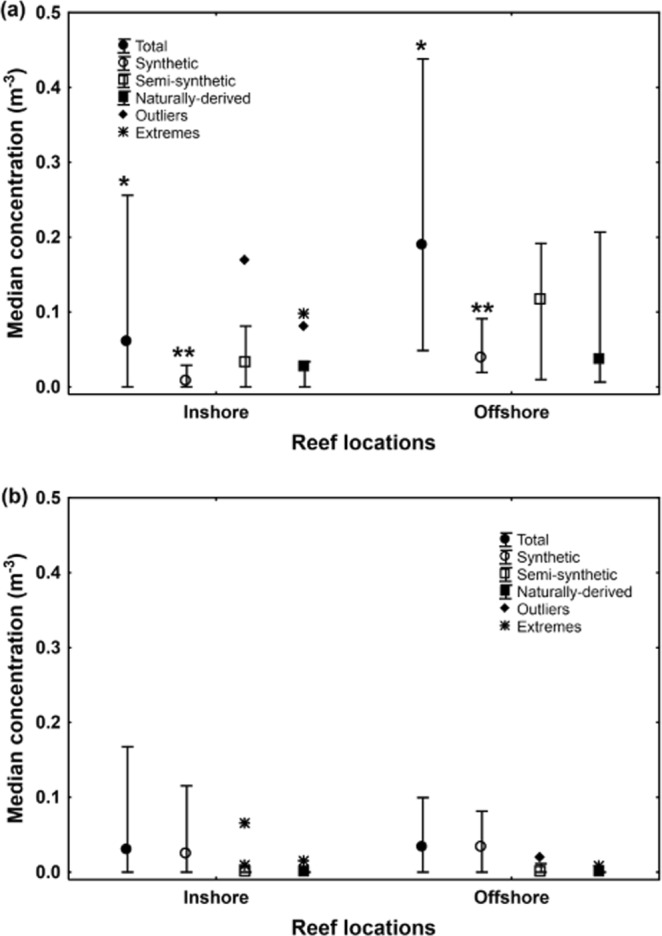
Concentrations of marine microdebris in surface water tows. Median concentration of marine microdebris, for (**a**) fibres and (**b**) particles, in 22 surface water tows conducted near inshore (n = 11) and offshore (n = 11) reef locations in the central region of the Great Barrier Reef World Heritage Area, Australia, 2016. Microdebris is classified as total, synthetic, semi-synthetic, and naturally-derived items. Whiskers show non-outlier range; * and **indicate significant differences using Mann-Whitney U-Test.

**Figure 5:**
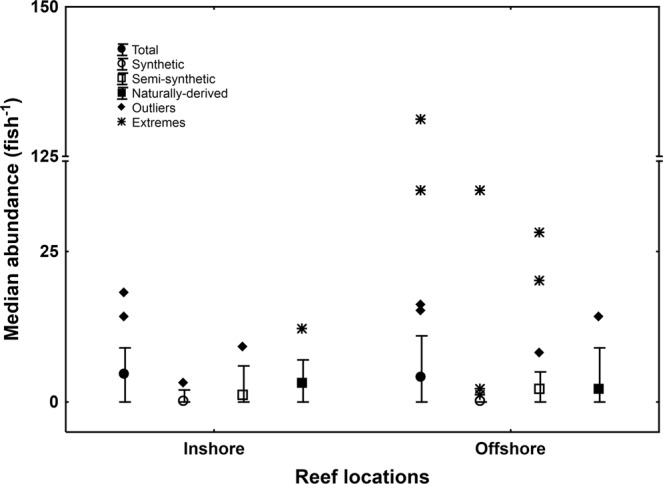
Abundance of marine microdebris items in Lemon damselfish. Median abundance of marine microdebris items in 60 Lemon damselfish, *Pomacentrus moluccensis*, collected at inshore (n = 30 fish) and offshore (n = 30 fish) reefs in the central region of the Great Barrier Reef World Heritage Area, Australia, 2016. Whiskers show non-outlier range; note that all (bar one particle from Davies reef, offshore) were fibres.

The abundance of marine microdebris differed between inshore and offshore reef locations for surface waters, but not for the microdebris ingested by Lemon damselfish. For surface waters, median concentration of total marine microdebris (fibres and particles) was almost double around offshore reefs (median = 0.25 m^−3^, range 0.05–0.47 m^−3^) compared to inshore reefs (median = 0.12 m^−3^, range 0.04–0.26 m^−3^), but this difference was not significant (Mann-Whitney U-test, U = 37, P = 0.13; Fig. S1a). More detailed analyses showed that the median concentration of total microfibres (U = 23, P = 0.015; Fig. 4a), and in particular synthetic microfibres (U = 2, P = 0.0001; Fig. 4a), but not total microparticles (U = 58.5, P = 0.92; Fig. 4b), were significantly higher in surface waters around offshore reefs compared to inshore reefs. This difference was not reflected in damselfish, with median abundance of total marine microdebris (fibres and one particle) not differing significantly between inshore (median = 4.5 fish^−1^, range 0–18 fish^−1^) and offshore (median = 4.0 fish^−1^, range 0–131 fish^−1^) reefs (U = 403.5, P = 0.50; Fig. 5). This lack of significant difference remained when one outlier (i.e. fish with 131 items) was removed from the analysis (U = 373.5, P = 0.36).

The most abundant colours of marine microdebris items were black, blue, white, and red, comprising ≥80% of the colours in both surface water tows and Lemon damselfish (Table 2). In addition, transparent fibres (i.e. lacking any colour) comprised 11% of all colours observed in damselfish, but were not detected in surface tows. The observed frequencies of microfibres with the most common colours (black, blue, white, transparent, red) in fish GIT differed significantly from those detected in surface waters (*Chi*-square = 277, df = 4, P < 0.0000001), due to white and transparent fibres being more frequently and blue and black fibres less frequently observed in fish GIT. In both surface waters and damselfish, marine microdebris items in remaining colours, such as brown, green, yellow and orange, were relatively uncommon and in total accounted for <10% of all items.

**Table 2:** Abundance (and proportion) of different coloured marine microdebris in Great Barrier Reef surface waters and Lemon damselfish.

Colour	Surface waters		Lemon damselfish	
	Fibres	Particles	Fibres	Particles
Black	209 (51)	3 (2)	147 (32)	0 (0)
Blue	117 (28)	28 (21)	54 (12)	0 (0)
White	47 (11)	64 (47)	149 (33)	0 (0)
Red	23 (6)	15 (11)	15 (3)	1 (100)
Blue + white	5 (1)	1 (1)	8 (2)	0 (0)
Brown	2 (0)	0 (0)	10 (2)	0 (0)
Green	2 (0)	8 (6)	3 (1)	0 (0)
White + orange	2 (0)	0 (0)	0 (0)	0 (0)
Yellow	1 (0)	1 (1)	6 (1)	0 (0)
Orange	1 (0)	1 (1)	9 (2)	0 (0)
White + red	1 (0)	0 (0)	0 (0)	0 (0)
Black + blue	1 (0)	0 (0)	0 (0)	0 (0)
Rusty	0 (0)	4 (3)	0 (0)	0 (0)
Transparent	0 (0)	7 (5)	49 (11)	0 (0)
Brown + white	0 (0)	0 (0)	0 (0)	0 (0)
Grey	0 (0)	4 (3)	0 (0)	0 (0)
Pink	0 (0)	0 (0)	2 (0)	0 (0)
Black + white	0 (0)	0 (0)	2 (0)	0 (0)
Total	411 (100)	136 (100)	454 (100)	1 (100)

Presence of marine microdebris was examined in 22 surface water tows and in the gastrointestinal tract of 60 Lemon damselfish, *Pomacentrus moluccensis*, collected near inshore and offshore reefs in the central region of the Great Barrier Reef World Heritage Area, Australia, in 2016.

**Figure 6:**
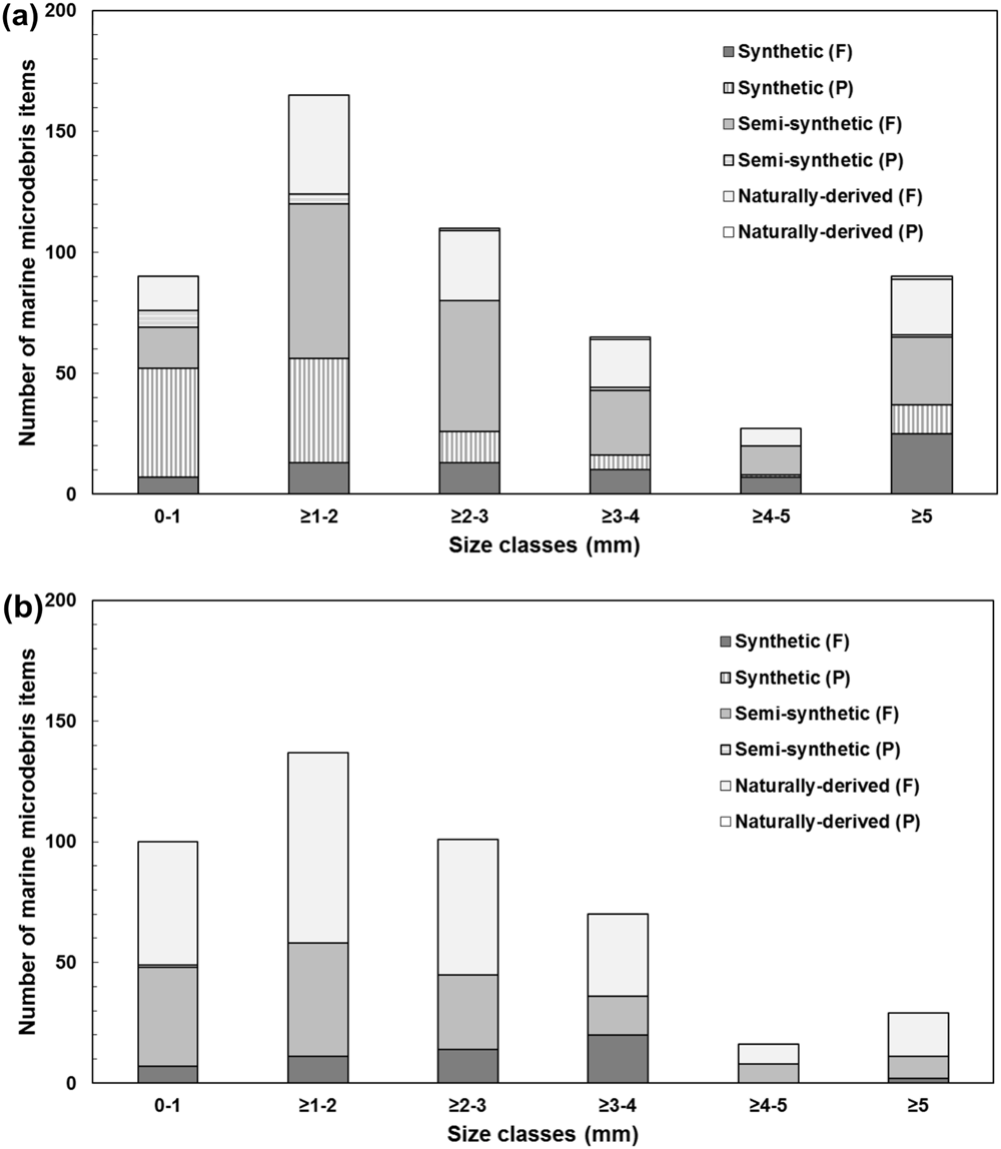
Size-frequency distributions of marine microdebris items. Marine microdebris items were detected in (**a**) all 22 surface tows, and (**b**) 57 of the 60 Lemon damselfish (*Pomacentrus moluccensis*) collected at inshore and offshore reef locations in the central region of the Great Barrier Reef World Heritage Area, Australia, 2016. F = fibre, P = particle.

The size distributions of marine microdebris in both surface waters and in Lemon damselfish were skewed towards the 0–3 mm size class, comprising 81% and 75% of the total items, respectively (Fig. 6a,b). Microplastics, i.e. items <5 mm confirmed by ATR-FTIR to contain plastic polymers, comprised just over half of the total marine microdebris detected in surface waters (n = 308, 56%), and only a quarter in Lemon damselfish (n = 111, 25%). Items ≥5 mm were more abundant in surface waters compared to in the GITs of Lemon damselfish, with maximum lengths of 55 mm and 13.1 mm, respectively. This included two pieces of fishing line, both detected in surface tows and chemically confirmed to be PE. Finally, the width of most fibres detected in surface waters (86%) and in Lemon damselfish (93%) was <50 µm, suggesting a textile origin^35–37^.

### Sources, transport and fate of marine microdebris

**Figure 7:**
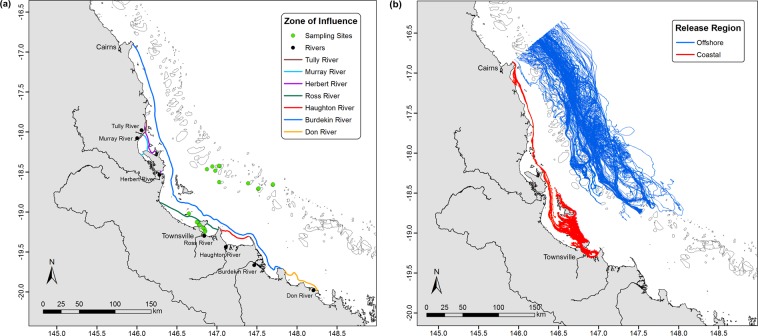
Numerical simulations of source, transport and fate of floating marine microdebris. Results of two numerical simulations conducted to examine (**a**) river discharge as a potential source of floating marine microdebris detected at the 22 sampling locations using passive tracers, and (**b**) potential transport pathways and fate of floating marine microdebris detected at the 22 sampling locations using virtual drogues. For the passive tracer study (**a**), the discharge points of the seven individual rivers are represented by black circles, and the 22 sampling locations are represented as green circles. Each colour represents the extent of the flood plume of individual rivers after a six-month simulation period up to 01 August 2016, For the virtual drogues study (**b**), trajectories in red are those from virtual drogues released at 11 inshore reef locations, and blue from 11 offshore reef locations; only every second release (i.e. every two minutes) is shown to avoid overexposure of and better visually define trajectories. The cessation of the trajectories (observed as a straight line) indicates that the drogues leave the numerical domain. For both numerical simulations, the modelling domain encompasses the study area where the collections were conducted (Figure S1).

Potential riverine sources of floating marine microdebris detected at the 22 sampling locations in the central GBR were examined using passive tracers in a numerical simulation for a six-month period up to 01 August 2016, encompassing the period during which surface tows were conducted (Fig. 7a). For the period simulated, the plumes of seven individual rivers that discharge into the central GBR are restricted to the coastal region and do not reach the outer reefs including the eleven offshore sampling locations. This includes the river plume from the Burdekin River, the largest of the seven river catchments considered here^38^, which discharges into the central GBR just south of Townsville. All eleven inshore sampling locations are within the zones of influence during the simulation period, suggesting that marine microdebris detected at these locations has the potential to be sourced from one or more of the seven coastal rivers. In contrast, all eleven offshore sampling locations are well outside the zone of influence during the simulation period, suggesting that the marine microdebris detected at these locations is not sourced from any of the coastal rivers within the six months simulation period.

Potential transport pathways and fate of floating marine microdebris detected at the 22 sampling locations in the central GBR were examined using virtual drogues in a numerical simulation for a 30-day period, starting on the day sampling was conducted at each of the 22 location (Fig. S3a,b; Fig. 7b). The simulated trajectories of particles derived from the hydrodynamic model released at the eleven inshore and eleven offshore sampling locations show clear differences 30 days after the sampling dates. Specifically, virtual drogues released from all inshore sampling locations present a cohesive and interrelated advection northwards (Fig. S3a). In contrast, virtual drogues released from offshore sampling locations initially present relative coherence only days after release, but subsequently more chaotic behaviour with a high degree of scattering over a larger region (Fig. S3b). The prevailing general direction for all virtual drogues was northwards (Fig. 7b). At no point, however, do transport trajectories from the eleven inshore sampling locations cross the central GBR lagoon to the offshore sampling locations, suggesting that marine microdebris detected at the offshore locations is unlikely to be sourced from inshore waters of the central GBR, based on the results from the limited (i.e. 30 day) simulation period.

## Discussion

To inform source reduction of marine microdebris contamination based on ecological effects, field studies are required to examine the interactions between microdebris exposure and intake by marine organisms. Our results demonstrate widespread contamination of the central GBR WHA with marine microdebris, including contamination of surface waters and intake in a common, small coral reef fish. These findings corroborate previous studies reporting microdebris contamination in GBR (sub-)surface waters^39,40^ and reef organisms^29,41^, indicating that contamination is ubiquitous across the GBR WHA. Marine microdebris was found in all 22 surface water tows, and in 57 of the 60 Lemon damselfish examined, with fibres (86%) being much more common than particles (14%) among the total 1,002 items detected. High abundance of fibres relative to particles in surface waters and marine fish has been reported previously in some^42,43^, but not all^7,44^ microdebris studies. Our findings show that marine microdebris in surface waters co-occur and interact with Lemon damselfish, however, the chemical composition, shape and colour of microdebris detected in fishes’ GIT contents differed significantly from that detected in surface waters. Specifically, Lemon damselfish contained relatively more semi-synthetic (e.g. NFPCs, rayon) and naturally-derived (e.g. cellulose) items, more fibres, and more white and transparent fibres, suggesting potential preferential feeding on, or avoidance of microdebris items with certain chemical cues and physical characteristics^45,46^. Riverine discharges were identified as a potential source for marine microdebris detected at inshore, but not at offshore reef locations, suggesting multiple sources^14,15^ contribute to contamination in the central GBR WHA. In contrast to some other studies^44,47^, primary microplastics such as microbeads from personal care products or pre-production plastic pellets were not observed.

Our estimated concentrations of marine microdebris in GBR surface waters range from 0.04 to 0.48 items m^−3^. These concentrations appear to be similar to those previously measured for microplastics in sub-surface waters of the central GBR^39^, but higher than those in surface waters of the northern GBR^10,40^. However, comparisons of reported concentrations for GBR marine waters, and international marine waters more broadly, are hampered by the use of different sampling, processing and analytical methods^10,22^. In the surface waters of the Pacific Ocean, for example, mean microplastic concentrations reported by 18 different studies vary by up to eleven orders of magnitude^10^. While some of these differences are undoubtedly due to the effects of oceanographic and environmental variables on microdebris distribution^10^, the relative effect of different methodologies on concentration estimates is likely to be large^25^. Methodological improvements that would aid in cross-study comparisons of marine microdebris contamination include (i) the standardisation of collection equipment such as net frame, size, mesh, and cod end design, (ii) consistency in microdebris processing, analyses, and classification, and (iii) uniform reporting metrics for microdebris concentration and abundance estimates^10,22,25,29^.

The detection and characterisation of marine microdebris is a rapidly emerging science underpinned by a fast developing methodology^48^. Our findings demonstrate the diversity of marine microdebris in the GBR environment, and highlight the importance of examining both chemical and physical characteristics to determine material origin and infer potential source material^22,23^. Applying a conservative workflow analysis to our samples^25,29^, we established that semi-synthetic and naturally-derived fibres were more common than synthetic particles in the GBR environment. The prevalence of fibres in marine microdebris is increasingly being acknowledged^10,15^, albeit often still erroneously reported as microplastics despite not always containing synthetic polymers^43,49^. Based on polymer type and fibre width most microfibres detected in our study were deemed to be of textile origin^35^, likely derived from clothing and furnishing^5,15^ rather than fishing lines or ropes^36,37^. Microdebris containing synthetic polymers comprised 60% of the items detected in surface waters, and a quarter of those in Lemon damselfish. Polyester and nylon were by far the most common polymer types detected in synthetic and semi-synthetic fibres, and are likely primarily sourced from the industrial textile sector^27^. Among particles, PE was the most common polymer type and is likely sourced from the industrial packaging sector^27^ through material such as plastic bags, bottles, food storage containers and plastic wrap^26^. Only two pieces were confirmed as PE fishing line, both in surface waters samples, supporting previous findings of relatively low abundance of fishing line in floating marine debris along Australia’s coastline^40^. Whether this reflects a true low abundance of fishing line in Australian marine debris, or an artefact of fishing-gear related plastics generally being negatively buoyant^26^ remains to be determined. Both polyester and PE are amongst the most commonly detected synthetic polymers in marine microplastics^5,7,18^, reflecting their large share in global plastic production and waste generation^27^. Future projections based on current plastic production and waste management trends suggest that the cumulative amount discarded in landfills or the natural environment will at least double from 2015 to 2050^27^. If these projections prove correct, marine microdebris contamination of the GBR WHA environment, and global marine environments in general, is going to be a long-term issue.

Land-based sewage effluent discharges have been posited as the main contributor to marine microfibre contamination^5,50^. Our study identified that riverine discharges are a potential source for marine microdebris detected in surface waters at inshore, but not at offshore reef locations. These results are in agreement with other studies on GBR hydrodynamics^51^ and riverine flood plume waters in the central GBR^52,53^. Specifically, the circulation in the GBR lagoon is dominated by tides and winds, including southeasterly trade winds during six months of the year^54^. Given that riverine flood plumes rarely reach offshore reefs even during extremely wet years^53^, and that wet season discharge from the seven coastal rivers in 2015–2016 was well below long-term medians^55^, marine microdebris detected at offshore locations is most likely derived from sources other than the coastal rivers examined. Indeed, our findings of higher concentrations in offshore waters combined with those from the numerical simulations strongly suggest that non-riverine sources are a major contributor to GBR marine microdebris contamination. These sources could range from (un-)intentional discard and sewage effluent discharges from vessels in the GBR^21^, to long-range atmospheric^56,57^ and oceanic^6,16^ transport. Recent reports indicate that atmospheric fallout could be a major contributor to marine microfibres contamination globally^15^, as evidenced by abundances of microdebris detected in Paris (between 2 and 355 particles m^−2^ day^−1^)^56^ and Dongguan (between 175 and 313 particles m^−2^ day^−1^)^57^. Airborne microdebris commonly originate from urban sources such as particles from road dust and fibres from textile furnishing^58^. The direction of the prevailing southeasterly trade winds^54^ would suggest that Australian urban sources are unlikely to directly contribute to atmospheric fallout on the GBR. The original sources from atmospheric fallout are likely to be extremely difficult to trace and predict^58^, and merit further investigation. Similarly, the contribution of riverine discharge to inshore microdebris contamination deserves further attention, as the concentration estimates for inshore waters are likely to be conservative given that wet season discharge prior to our study was well below long-term medians^55^.

Distribution patterns of microdebris in marine environments are influenced by their chemical and physical characteristics in combination with environmental factors. The clear differences in virtual drogue behaviour released from inshore and offshore locations are intrinsically linked to the hydrodynamics of the GBR, which is strongly influenced by the bathymetric complexity of the region^51^. Inshore, this behaviour stems from the bathymetric smoothness and the absence of a coral reef matrix system in the coastal region^51^. Offshore, this behaviour is influenced by the complex bathymetry, and associated currents of the outer coral reef matrix^51^. The prevailing general direction for all virtual drogues was northwards which is consistent with previous studies^54^. Inclusion of the relative density, drag coefficient and persistence in numerical simulations would provide further detail on horizontal and vertical transport pathways^19,59^. For example, low-density materials such as PE are more likely to float and disperse more widely in surface waters than high-density materials such as polyvinyl chloride^7^. Moreover, naturally-derived and semi-synthetic items are less likely to persist in the marine environment than synthetic items^26,60^, although in tropical environments with high UV-radiation the deterioration rate of synthetic items may be significantly increased^61^. Indeed, once in the marine environment both chemical and physical characteristics are affected by abiotic (e.g. UV-radiation, oxygen) and biotic (e.g. microbial, fouling) degradation^26,60^, influencing the drift trajectories and distribution patterns of microdebris^19^. Future research on these processes is critical to estimate how long microdebris has been in the marine environment, estimate drift trajectories, and identify potential source regions. Moreover, such information will also assist in elucidating the ultimate fate of microdebris items in the marine environment, whether this is through further fragmentation, settlement onto benthic substrates, or intake into food webs.

The intake of marine debris by marine fish could be a result of direct ingestion, or indirect ingestion via their food sources. Lemon damselfish have been described as planktivorous^62^ and omnivorous^63^, with our observations on gut content supporting the latter. The intake of microdebris by damselfish appears to be non-random, with chemical composition, shape and colour differing significantly from that detected in surface waters. Whether this reflects preference or avoidance behaviour based on chemical^45^ or visual^46^ cues is currently unknown. The higher observed frequency of fibres, and specifically of white and transparent fibres, in damselfish may reflect their similarity to their main food items such as calanoid copepods and benthic algae^63^. The prevalence of white plastics, including microdebris in fish gut contents has been reported in some^29,64,65^, but not all studies^66,67^. The presence of transparent fibres, comprising synthetic, semi-synthetic, and naturally-derived materials, is more difficult to explain given their complete lack in surface waters, but may be a result of fibres losing their colourants following exposure to digestive fluids during gut retention. In this and other studies^29,67,68^, the total number of ingested microdebris items did not increase with fish size, indicating that accumulation is ephemeral and most if not all ingested items are egested^69^. Indeed, for most of the wild-caught fish species examined so far their condition seemed not to be affected by the amount of ingested microdebris^29^. Our findings highlight the importance of including fibres in estimates of microdebris contamination, as intake of man-made items by Lemon damselfish would otherwise incorrectly have been assessed as non-existent. Whether the prevalence of cellulose and rayon fibres in both damselfish and coral trout (*Plectropomus leopardus*)^29^, one of its predators, reflects food web transference of marine microdebris remains to be confirmed.

Given that marine debris contamination is projected to increase in the future^27^, our work outlines a robust approach towards increasing our understanding of the source and distribution of microdebris and the interactions with marine organisms. Applying a conservative workflow analysis to our surface water and damselfish samples^25,29^, our findings demonstrate the diversity in origin, distribution and characteristics of marine microdebris contamination in the GBR environment. These findings should be taken into account in future exposure experiments designed to determine the effects of contamination on marine organisms and ecosystems, by including microdebris types and concentrations^70^ that are environmentally realistic. As it is unlikely that marine microdebris contamination is amenable to clean-up efforts^4^, it is critical for the next generations of simulation models to be able to resolve riverine and non-riverine sources and include associated processes such as marine debris degradation^59^. Combined with improved understanding of interactions between marine organisms and microdebris contamination, this will contribute to informing source reduction based on ecological effects.

## Materials and Methods

### Study area

The study area is located in the central region of the GBR WHA, Australia (Fig. 1). The tropical climate in this region is characterised by marked wet summers (November to April) and dry winters (May to October), with the majority of total rainfall (650 to ≥1,200 mm) occurring from January to March (http://bom.gov.au). Surface tows and fish collections were conducted at locations near inshore reefs (i.e. reefs 1–30 km from the mainland) and offshore reefs (i.e. reefs 60–100 km from the mainland; Table S1). The inshore and offshore locations are geographically separated by the GBR lagoon, with inshore reefs closer to riverine and urban discharges while offshore reefs are more exposed to oceanic conditions. Depths range from <15 m around inshore reefs to ~50 m around offshore reefs.

### Collection of surface water samples and coral reef fish

The presence and abundance of marine microdebris was examined in surface waters and in Lemon damselfish (*Pomacentrus moluccensis*) at both inshore and offshore reefs (Fig. 1). Twenty-two surface tows were conducted at eleven inshore and eleven offshore locations during three trips aboard the AIMS Research Vessel (RV) Cape Ferguson in April–July 2016 (Table S1), following procedures detailed in Kroon *et al*.^25^. Briefly, the air-sea interface was sampled using a plankton net (355 µm polyester (PES) mesh) with a 750 mL clear collection jar (PP); Investment Co.) as cod-end. Tows were conducted for approximately 10 min (mean 10.1 min ± 0.1 s.e.m., n = 22) in a straight line with half of the rectangular frame submerged in the surface water. Proper frame positioning was maintained by vessel speed (mean 1.4 kn ± 0.1 s.e.m., all tows <4.4 kn, n = 22), the length of the hydraulic winch cables, and weights (5 kg) attached to the lower edge of the frame. To estimate tow length, Global Positioning System (GPS) coordinates were recorded at the start and finish of each tow using a handheld GPS instrument (Garmin GPSMAP 78) (mean tow length 1.2 km ± 0.06 s.e.m., n = 22). On average, each tow covered a sea surface area of 900 m^2^ ± 46.2 s.e.m. (n = 22), and filtered a sea water volume of 135 m^3^ ± 6.9 s.e.m. (n = 22). Following retrieval back on the vessel, the plankton net was rinsed down from the outside with seawater into the cod-end, and volume-reduced through a 37 µm nylon mesh. Each sample was rinsed with filtered (37 µm) desalinised (<1 µm) seawater into a 50 mL vial (PP clear cup, high density PE yellow screw cap; Sarstedt), preserved in 70% ethanol (EtOH, prepared from absolute with filtered (37 µm) desalinised (<1 µm) seawater), and stored cold at 11 °C until further processing.

Sixty Lemon damselfish were collected at three inshore reefs (n = 30) and two offshore reefs (n = 30) between June and October 2016 during four trips on board RV Cape Ferguson and RV Apollo (Table S1), following procedures that complied with all relevant ethical regulations (Great Barrier Reef Marine Park Authority permit G12/35236.1 and James Cook University Animal Ethics Committee Approval Number A2293). The collection reefs were located within the vicinity of surface water sampling locations (Fig. 1). Lemon damselfish are an obligate coral-dwelling species^71^ common to shallow Indo-Pacific coral reefs^33^. It has been described as planktivorous obtaining food particles carried on water currents^62^, and as omnivorous feeding primarily on calanoid copepods and benthic algae^63^. The species has a small home range of a few square meters^33^ suggesting that any intake of marine microdebris reflects local exposure. Fish were captured on SCUBA at 1–12 m depth using a fence net and diluted clove oil (20% clove oil: 20% EtOH: 60% filtered (37 µm) desalinised (<1 µm) seawater^72^. Fish were euthanized immediately with an overdose of clove oil and placed in individual resealable bags. Fish were frozen immediately upon return to the vessel and stored at −20 °C until further processing.

### Sampling of fish GIT contents

Fish were defrosted, measured (total length; TL, in mm), weighed (W, in g) (Mettler Toledo, PL403), and dissected. For each individual fish the entire GIT was removed from the top of the oesophagus to the rectum as per Kroon *et al*.^29^, with the following slight modifications. Dissection was conducted under a stereomicroscope (Leica M26) and GIT contents filtered through a 37 µm mesh. The GIT contents were placed in a Bogorov chamber, covered with aluminium foil to prevent loss of items and introduction of contaminants, until further processing. The GIT itself was placed in a glass petri dish and processed immediately.

### Processing and analyses of potential marine microdebris items

To identify, characterise and quantify potential marine microdebris items in surface water samples and in fish GIT contents, the workflows outlined in Kroon *et al*.^25,29^ were followed. For surface water samples, each individual sample was filtered through a 37 µm mesh and placed in a Bogorov chamber. Each individual surface water and fish GIT sample was visually examined in the Bogorov chamber under a digital stereomicroscope (Leica M165C, 0.73 x –12.0x magnification). Potential marine microdebris items were individually selected using metal needle nose forceps or a hooked microneedle and placed onto concave glass slides. Following exhaustive visual examination, the concave glass slide was covered with a flat glass cover slide, sealed with tape to prevent loss of items and introduction of contaminants, and dried at 60 °C for 24 hours. Using a HD Digital microscope camera (Leica MC170 HD), all individual items were subsequently (i) photographed to enable further visual inspection, (ii) measured to establish length (0.001 mm, length = longest axis) for size distribution analyses, and (iii) measured to establish width (0.001 mm; fibres only) to distinguish between textile (≤50 µm) and other sources such as fishing material (>50 µm)^35–37^. All measurements were done using the image processing program ImageJ (LAS V4.4).

The chemical composition of individual items was examined by FTIR (Perkin-Elmer Spectrum 100 FTIR spectrometer operating with Spectrum Software V11.0) using ATR in transmission mode, as described by Kroon *et al*.^25,29^ with minor modifications: 4 scans at 4 cm^−1^ resolution, wavenumber range 4,000–650 cm^−1^ and background scans acquired after every 20^th^ sample. Spectra were baseline corrected and searched (3,900-650 cm^−1^ region; the atmospheric water/CO_2_ region between 2,500 and 1,900 cm^−1^ was excluded) against commercially available Nicodom IR spectral libraries (Polymers and Additives, Coatings, Fibres, Dyes and Pigments, Petrochemicals; Nicodom Ltd., Czech Republic) to give a percent match between the sample and the reference spectrum. To ensure a high level of confidence in chemical characterization of individual items, only those with a high level of certainty (i.e. match between ≥60 and 100%)^25,43^ were initially accepted for further examination. All spectra were subsequently closely inspected and eliminated if key diagnostic signals were missing^25^.

All items that passed spectral interpretation and interrogation were then assessed against a customised contaminant library developed specifically for this study (see ‘Preventing contamination of samples’). Physical characteristics of separated items with percent match of ≥90% to one or more contaminants were further inspected, specifically size, shape, texture, and colour, and compared to those of the known items in the contaminant library. Items were examined conservatively, and if contamination could not be ruled out due to similarities in physical characteristics, the item was considered to have been introduced during collection, processing and/or analyses, and excluded from further analyses.

Following the contamination check, spectra of all remaining items were interrogated against each other using the PerkinElmer Compare algorithm to validate chemical assignment. Subsequently, the likely source (human or natural) was established through further visual inspection of photographs for physical characteristics following Kroon *et al*.^25,29^. Again, items were examined conservatively, and if a natural origin could not be ruled out the item was assigned as natural and excluded from further analyses. The remaining marine microdebris items were considered to have been manufactured, modified or used by humans, and were further classified as synthetic, semi-synthetic, and naturally-derived as per Kroon *et al*.^29^:

- **Synthetic**: items manufactured by chemical synthesis, including through the process of polymerization. This includes thermoplastics (e.g. nylon, PE, PP, and PS), and thermoset and elastomer plastics (e.g. polyester, polysiloxane, and polyurethane).
- **Semi-synthetic**: items manufactured synthetically from one or more substances of natural origin. This includes materials regenerated from one or more natural substances (e.g. rayon derived from cellulose), and materials that are composites of natural and synthetic substances (i.e. natural fibre reinforced polymer composites, NFPC^34^).
- **Naturally-derived**: items manufactured from one or more substance of natural origin. This includes materials derived from plants (e.g. cotton, flax, hemp, linen, ramie) and animals (e.g. wool, fur), and from inorganics (e.g. calcium carbonate, calcium silicate). Materials that are composites of two or more natural substances (e.g. mixed yarns from natural fibres) are also included here.

### Preventing contamination of samples

To prevent contamination, exposure time of samples was minimised during collection, processing and analyses following Kroon *et al*.^25,29^, and as recommended by Woodall *et al*.^73^. Briefly, field samples were processed and preserved immediately following collection. Samples of vessel-based water used during on-board processing were examined for possible contamination by stereomicroscopy, including water from a deck hose used for rinsing the outside of plankton nets, and desalinised (<1 µm) tap water used for rinsing laboratory equipment and making up 70% EtOH solution. During on-board processing and in the laboratory, four glass petri dishes filled with desalinised (<1 µm) tap water (on-board), or with Milli-Q water (MQ H_2_O, 18.2 MΩ.cm at 25 °C; laboratory) were placed adjacent to the work areas. These were later analysed as procedural blank controls to check for inadvertent airborne contamination. Prior to processing on the vessel and in the laboratory, hands and forearms were washed and clothing was rolled with a lint-roller, and all work surfaces, glassware, dissection tools, and other equipment were cleaned with 70% EtOH and checked for contamination before use as well as in-between individual samples^25,74^. Dissection of fish GIT and subsequent visual separation of potential marine microdebris items from these, and from surface tow samples were conducted in a secure microscope room. When not in direct use, all glassware and samples were covered with glass covers or aluminium foil^73,74^. The diamond head of the ATR-FTIR was cleaned with methanol and lint-free tissue followed by visual inspection (using a 2x magnification MAGGYLAMP) between each item. Reference samples of materials (n = 26) from surface tow collection (RV Cape Ferguson: deck ropes (green and white); paint and rust chips; plankton net mesh, canvas, and cod end), surface tow field processing (lint-free tissue; 37 µm white mesh; 50 mL vial clear base and yellow lid; brown rubber bands), fish collection (clove oil bottle; fence (transparent) and hand nets (blue and green); resealable bag), laboratory processing (blue and green nitrile gloves; brown rubber bands; white tape; red microscope cover; transparent Bogorov chamber), and ATR-FTIR (laboratory coat) were retained for physical and chemical characterisation^25,74^ (Table S3). The ATR-FTIR spectra of these reference samples were collated in two customised contaminant libraries (one for surface tow samples, one for fish GIT samples) developed specifically for this study.

### Data analyses

Data analyses were conducted on items considered to be marine microdebris. First, for each individual surface tow sample and individual fish sample, the number of marine microdebris items classified as synthetic, semi-synthetic or naturally-derived was quantified and categorised according to main chemical type. To examine potential preferential feeding on marine microdebris items with specific characteristics, their *expected* frequency (in all surface tow samples) was compared with their *observed* frequency (in all fish GIT samples) using *Chi*-square test. Specific characteristics thus examined were (i) categories (i.e. synthetic, semi-synthetic and naturally-derived items), (ii) chemical types, and (iii) colours. Second, for each surface tow sample, the concentration of marine microdebris items (*C*_*s*_, particles m^−3^) was estimated at each sampling location^10^, by calculating the volume of seawater filtered based on tow distance and frame dimensions following Kroon *et al*.^25^. To examine potential differences in abundance of marine microdebris items between inshore and offshore reef locations, two-tailed Mann-Whitney U tests were conducted for surface tow samples and for fish GIT samples, respectively. Finally, to determine the relative contamination of microplastics as a proportion of total number of marine microdebris items detected in surface tow samples and in fish GIT samples, respectively, the size frequency distributions of synthetic, semi-synthetic and naturally-derived items were plotted. Nonparametric tests were used when assumptions of normality and homogeneity of variance could not be met^75^. Tests of significance are two-tailed unless otherwise stated. Statistical analyses were conducted in Statistica^76^.

### Numerical simulations

To improve understanding of potential sources, transport and fate of marine microdebris detected in surface waters of the central GBR, two numerical simulations were conducted with a hydrodynamic model using the Delft3D integrated modelling suite^77^ (for more detail, see Supplementary Information). Briefly, the first numerical simulation was designed to test the hypothesis that marine microdebris detected at the 22 sampling locations could have originated from one or more of the seven rivers discharging into the central GBR. In this simulation the zone of influence of individual rivers plumes was established using river-tagged passive tracers for a six-month period (01 February–01 August 2016), encompassing the period during which surface tows were conducted (April–July 2016; Table S1). The second numerical simulation was designed to examine potential transport pathways and fate of floating marine microdebris detected at the 22 sampling locations, had they not been removed from their sampling locations. Virtual drogues, representing floating marine microdebris, were released at the surface from the location, on the date and around the time of the respective sampling occasion and their trajectories simulated for 30 days using corresponding hydrodynamic parameters. A density of 1 g cm^−3^ (equivalent to fresh water) was assigned to the virtual drogues; specific densities of the marine microdebris items most commonly detected in our sea surface samples could not be considered due to constraints on computer processing time. For both numerical simulations, the modelling domain encompasses the study area where the collections were conducted (Fig. S2).

## Supplementary information

Supplementary File

## Data Availability

Data are available from the corresponding author on request.
