# Supplementary material for: Sources, distribution and fate of microfibres on the Great Barrier Reef, Australia
Source: Sci Rep. 2019 Jun 21;9:9021. doi: 10.1038/s41598-019-45340-7 (PMC6588688; doi:10.1038/s41598-019-45340-7)
Supplement: Supplementary file 1 — Supplementary File [file 41598_2019_45340_MOESM1_ESM.docx]

**Sources, distribution and fate of microfibres on the Great Barrier Reef, Australia**

Lene H. Jensen^1,2^, Cherie A. Motti^1^, Anders L. Garm^2^, Hemerson Tonin^1^ & Frederieke J. Kroon^1,*^

^1^ Australian Institute of Marine Science, Townsville, Qld 4810, Australia

^2^ University of Copenhagen, Universitetsparken 4, 2100, Copenhagen, Denmark

* Correspondence and requests for materials should be addressed to F.J.K. (email: f.kroon@aims.gov.au)

Supplementary Information

# Text

## Analyses of fish length

The 60 fish from the five study locations had an average total length (TL) of 59 mm (± 9 standard deviation, s.d.), with a size range between 30 and 75 mm (Table S1b). The average fish weight was 4.9 g (± 2.2 s.d.), ranging from 0.6 to 10.9 g. Fish collected at inshore and offshore locations differed in weight and length, with fish from two inshore reefs (Magnetic and Herald Island) being significantly shorter than fish from both offshore reefs (two-tailed One-way ANOVA, F_4,55_ = 7.9, P<0.001, followed by Tukey-Kramer post hoc test, 0.001<P<0.004). Fish from Magnetic Island weighed less than those from the offshore reefs, similarly fish from Herald Island were lighter than the fish from John Brewer Reef (two-tailed One-way ANOVA, F_4,55_ = 4.8, P=0.002, followed by Tukey-Kramer post hoc test, 0.044>P>0.01). Fish from Great Palm Island did not differ significantly from those at any of the other reefs (00.13<P<0.86).

## Numerical modelling simulations

Two numerical simulations using a hydrodynamic model were conducted using the Delft3D integrated modelling suite ^1^. Delft3D was selected because it (i) contains a number of features relevant to the marine microdebris found in the surface water tow samples (e.g. precise geographical release location, release and recovery time, and specific drag conditions), and (ii) includes several modules that have tools for pre- and post-processing that facilitates the implementation and analyses of its output.

The numerical simulation domain encompasses the approximately 100 x 100 km sampling area off Townsville, and extends at least 200 km north, east and south past its boundaries (Fig. S1). The numerical grid has horizontal dimensions, 451 x 247 grid points, with a spatial resolution of 1 km, and one vertical layer (i.e. 2D simulations). The bathymetric dataset implemented in this grid was obtained from the digital elevation model of the GBR produced at 100 m spatial resolution ^2^, and bathymetric data were associated to each grid point by triangular interpolation. The sea surface elevation used in the model’s boundaries is based on a comprehensive modelling formulation, including barotropic (i.e. tides), baroclinic (i.e. salt, temperature and water pressure) and atmospheric components (i.e. wind, pressure, rain, evaporation). Specifically, the oceanic open boundaries were forced with the results of the Sparse Hydrodynamic Ocean Code ^3^, as implemented in the eReefs project ^4, 5^. The model applied in the eReefs project has a spatial resolution of the order of 4 km; to reach the spatial resolution of 1 km in our study the boundaries were forced by time series of current, elevation, temperature and salinity, and linearly interpolated. For atmospheric forcing we used the winds fields and the atmospheric pressure, at a height of 10 m above sea level, obtained from the ACCESS meteorological model run operationally by the Australian Government Bureau of Meteorology (http://www.bom.gov.au/nwp/doc/access/NWPData.shtml). The river discharges were forced using time series of the volumetric discharge and ambient temperature obtained from the Queensland Government Department of Natural Resources and Mines stream gauging network (http://watermonitoring.dnrm.qld.gov.au/host.htm). The river discharges into the central GBR was considered with salinity equalling zero (i.e. fresh water). Overall, the entire methodology for the forcing used in this numerical simulation remained consistent with the eReefs project ^4, 5^, both in relation to the origin of the data and in the methodology of use in the numerical model.

Following extensive simulation to establish the energy equilibrium and distribution of currents, temperature and salinity field consistent with those obtained by eReefs ^4, 5^, the hydrodynamic model was considered validated and suitable for the two numerical simulations conducted in this study.

### Riverine sources of marine microdebris detected in the central GBR.

First, we examined whether the marine microdebris detected in surface waters at the 22 sampling locations could have originated from one or more of the seven rivers discharging into the central GBR (Don, Burdekin, Haughton, Ross, Herbert, Murray and Tully). The zone of influence of each individual river was determined by releasing river-tagged passive tracer from each of the seven rivers on 01 February 2016, and following them for six months to 01 August 2016. This period encompassed the sampling period (April-July 2016; Table S1a), and includes three months prior to the first surface water tow conducted on 30 April 2016. Passive tracers act as virtual markers and do not interfere in the hydrodynamic circulation nor interact with each other; the only processes occurring being diffusion and advection. This methodology enables the identification of the destination of each of the river plumes individually; note that a receiving water body can receive water from more than one river.

### Transport and fate of marine microdebris detected in the central GBR.

Next, we examined potential transport pathways and fate of floating marine microdebris detected in surface waters at the 22 sampling locations in the central GBR, had they not been removed from their sampling locations. Virtual drogues were released on the surface from the location and date of the respective sampling occasion and followed for 30 days. Specifically, at each of the 22 sampling locations 60 virtual drogues were released at one minute intervals for one hour, starting 30 minutes prior to the start of the real surface water tow. This numerical exercise was designed based on the analogous behaviour of numerical drogues and floating marine debris. Specific abundance, size or densities of the different marine debris items most commonly detected in our study were not considered in the virtual drogues due to constraints on computer processing time. Note that both the date and time of the release of virtual drogues thus differs across the 22 sampling locations, as well as the date and time when they are no longer considered in this numerical simulation.

## Processing and analyses of potential marine debris items

### Surface water tows

The number of potential marine debris items included and excluded from the analyses is presented for each step of the workflow applied to the 22 tow samples (Table S2a). Potential marine debris items (n=681), comprising both fibres (n=494, 73%) and particles (n=187, 27%), were separated from all 22 surface water tows following visual examination under a stereomicroscope. A total of 628 individual items (470 fibres, 158 particles) were analysed by ATR-FTIR spectroscopy. The discrepancy between the number of items separated (681) and analysed (628) was due to items being lost during transfer from glass slide to ATR-FTIR diamond head, or being too small to place confidently on the ATR-FTIR diamond head. Of the 628 individual items, ten (2%) items presented a match of <60% and were not considered for further analyses. A further 18 items returned a match of between 60 - <70%, and a total of 80 items showed a match of between 70-<80%. Key diagnostic signals were not detected for 14 of these items, likely due to co-occurrence with other components such as protein. Comparative analyses of the spectra of these 14 items against all 628 spectra resulted primarily in matches with other items that had similar spectral matches. Given the low confidence in their ATR-FTIR spectra and a lack of diagnostic chemical signals, these 14 items were subsequently excluded from further analyses. Hence, of the 628 items initially analysed by ATR-FTIR, a total of 24 (10+14) were eliminated due to poor matches to the Nicodom IR libraries and the lack of diagnostic chemical signals in their spectra. A total of 604 individual items (454 fibres, 150 particles) were included in further analyses (Table S2a).

During processing and analyses of surface water tow samples on the RV Ferguson and in the AIMS laboratory, a total of 16 fibres were found in petri dishes placed adjacent to the work area (Table S3a). In addition, reference samples of materials used during collection and processing (n=20) were retained (Table S3a). The structure, shape, texture and colour of these items were noted, their chemical composition analysed by ATR-FTIR spectroscopy, and their spectra added to the customised contamination library for surface water tow samples. Spectral comparison of the 604 potential marine debris items with those in the customised contaminant library revealed that 162 items showed a ≥90% chemical correlation to one or more known contaminants (Table S2a). These matches were primarily to spectra from laboratory coat fibres (Table S4a). Visual inspection and comparison of the physical characteristics of these 162 items with those of the actual items entered in the customised contaminant library, showed that 19 items showed similarities in both spectra (i.e. ≥90% match) and physical characteristics (i.e. shape, texture, and colour). These 19 items could not be excluded as potential contaminants in the samples and were therefore not considered in any further analyses (Table S2a). The remaining 585 items (435 fibres, 150 particles) were considered for further analyses (Table S2a).

Based on ATR-FTIR and subsequent spectral interrogation, the chemical composition of 259 of the remaining 585 individual items (231 fibres, 28 particles) indicated they were potentially of natural origin. Closer inspection of their physical characteristics (i.e. structure, shape, texture and colour) revealed that a natural origin of a total of 38 items could not be excluded. Thus, a final total of 547 out of the 585 items were assigned to be marine debris (Table S2a), comprising 80% of the 681 potential marine debris items that were first visually separated using stereomicroscopy.

### Damselfish

The number of potential marine debris items included and excluded from the analyses is presented for each step of the workflow applied to the 60 individual fish (Table S2b). Potential marine debris items (n=556), comprising both fibres (n=546, 98%) and particles (n=10, 2%), were separated from 58 fish following visual examination under a stereomicroscope. A total of 503 individual items (495 fibres, 8 particles) were analysed by ATR-FTIR spectroscopy. Similar to the surface water tow samples, the discrepancy between the number of items separated (556) and analysed (503) was due to items being lost during transfer from glass slide to ATR-FTIR diamond head, or being too small to place confidently on the ATR-FTIR diamond head. Of the 503 individual items, six (1%) items had a match of <60% and these were not considered for further analyses. A total of nine items showed a match of between 60 - <70%, and a total of 36 items showed a match of between 70-<80%. Key diagnostic signals were not detected for eight of these items, respectively, likely due to co-occurrence with other components such as protein. Comparative analyses of the spectra of these eight items against all 503 spectra resulted primarily in matches with other items that had similar spectral matches. Given the low confidence in their ATR-FTIR spectra and a lack of diagnostic chemical signals, these eight items were subsequently excluded from further analyses. Hence, of the 503 items initially analysed by ATR-FTIR, a total of 14 (6+8) were eliminated due to poor matches to the Nicodom IR libraries and the lack of diagnostic chemical signals in their spectra. A total of 489 individual items (481 fibres, 8 particles) were included in further analyses (Table S2b).

During the dissection and processing of damselfish in the AIMS laboratory, a total of ten fibres were found in petri dishes placed adjacent to the work area (Table S3b). In addition, reference samples of materials used during collection and processing (n=15) were retained (Table S3b). The structure, shape, texture and colour of these items were noted, their chemical composition analysed by ATR-FTIR spectroscopy, and their spectra added to the customised contamination library for fish samples. Spectra comparison of the 489 potential marine debris items with those in the customised contaminant library revealed that 61 items showed a ≥90% chemical correlation to one or more known contaminant (Table S2b). As for the surface water tow samples, these matches were primarily to spectra from laboratory coat fibres (Table 4b). Visual inspection and comparison of the physical characteristics of these 61 items with those of the actual items entered in the customised contaminant library, showed that 14 items showed similarities in both spectra (i.e. ≥90% match) and physical characteristics (i.e. shape, texture, and colour). These 14 items could not be excluded as potential contaminants in the samples and were therefore not considered in any further analyses (Table S2b). The remaining 475 items (467 fibres, 8 particles) were considered for further analyses (Table S2b).

Based on ATR-FTIR and subsequent spectral interrogation, the chemical composition of 256 of the remaining 475 items (249 fibres, 7 particles) indicated they were potentially of natural origin. Closer inspection of their physical characteristics (i.e. structure, shape, texture and colour) revealed that a natural origin of a total of 20 items could not be excluded. Thus, a final total of 455 out of the 475 items were assigned to be marine debris (Table S2b), comprising 80% of the 566 potential marine debris items that were first visually separated using stereomicroscopy.

# Tables

**Table S1**. **Sampling locations**. Locations of (a) surface water tows near, and (b) fish collections at inshore and offshore reefs of the central Great Barrier Reef World Heritage Area, Australia, conducted in 2016. For surface water tows, the date, start position (latitude, longitude), duration (min), surface area (m^2^) and volume (l) for each tow is given. For fish collections, the number collected at each site (*n*), and measurements of total length (TL, mm) and weight (W, g) are given, including s.d. = standard deviation, Min = minimum, and Max = maximum.

(a)

| **Number** | **Date** | **Location** | **Start position** | **Duration (min)** | **Surface area (m^2^)** | **Volume (m^3^)** |
| --- | --- | --- | --- | --- | --- | --- |
|  |  |  | **(latitude, longitude)** |  |  |  |
| Offshore |  |  |  |  |  |  |
| 1 | 30.04 | Kelso reef | 18°25.885'S, 146°56.684'E | 11.42 | 1,739 | 261 |
| 2 | 01.05 | Roxburgh reef | 18°25.596'S, 147°02.086'E | 10.00 | 873 | 131 |
| 3 | 02.05 | Fore and Aft reef | 18°28.969'S, 147°01.605'E | 10.03 | 1,058 | 159 |
| 4 | 07.05 | Chicken reef | 18°39.638'S, 147°41.985'E | 10.08 | 737 | 111 |
| 5 | 07.05 | Grub reef | 18°38.473'S, 147°23.448'E | 09.25 | 955 | 143 |
| 6 | 10.05 | Little Kelso reef | 18°28.990'S, 146°58.799'E | 10.16 | 844 | 127 |
| 7 | 11.05 | Rib reef | 18°27.982'S, 146°52.527'E | 10.06 | 688 | 103 |
| 8 | 13.05 | Centipede reef | 18°42.638'S, 147°30.927'E | 10.07 | 918 | 138 |
| 9 | 16.05 | Chicken reef | 18°39.275'S, 147°42.062'E | 10.02 | 598 | 90 |
| 10 | 17.05 | Centipede reef | 18°42.362'S, 147°31.404'E | 10.13 | 730 | 110 |
| 11 | 19.05 | John Brewer reef | 18°37.559'S, 147°01.766'E | 10.04 | 807 | 121 |
| Inshore |  |  |  |  |  |  |
| 12 | 23.06 | Ross River | 19°14.345'S, 146°51.291'E | 10.40 | 903 | 135 |
| 13 | 23.06 | #25 | 19°12.872'S, 146°50.749'E | 10.44 | 984 | 148 |
| 14 | 23.06 | Middle reef | 19°12.038'S, 146°49.688'E | 10.18 | 910 | 137 |
| 15 | 23.06 | West of Magnetic Island (1) | 19°10.071'S, 146°47.091'E | 10.05 | 910 | 137 |
| 16 | 23.06 | West of Magnetic Island (2) | 19°09.189'S, 146°46.513'E | 10.16 | 925 | 139 |
| 17 | 23.06 | West of Magnetic Island (3) | 19°09.229'S, 146°46.632'E | 10.07 | 999 | 150 |
| 18 | 23.06 | Cockle Bay Reef | 19°09.700'S, 146°47.127'E | 10.00 | 829 | 124 |
| 19 | 16.07 | Ross River mouth | 19°14.894'S, 146°51.070'E | 10.32 | 925 | 139 |
| 20 | 16.07 | #39 | 19°07.529'S, 146°45.075'E | 10.27 | 873 | 131 |
| 21 | 16.07 | Rattlesnake | 19°01.173'S, 146°39.080'E | 09.54 | 755 | 113 |
| 22 | 16.07 | Palm Island | 18°47.068'S, 146°27.360'E | 10.29 | 836 | 125 |

(b)

| **Number** | **Date** | **Location** | **Position (latitude, longitude)** | ***n*** | **Size (mm, TL)** | |  | **Weight (g)** | | |
| --- | --- | --- | --- | --- | --- | --- | --- | --- | --- | --- |
|  |  |  |  |  | **Mean ± s.d.** | **Min** | **Max** | **Mean ± s.d.** | **Min** | **Max** |
| Inshore |  |  |  |  |  |  |  |  |  |  |
| 1 | 17.-18.06 | Great Palm Island | 18°42.105'S, 146°34.640'E | 8 | 58.4 ± 5.5 | 51 | 66 | 4.5±1.5 | 2.7 | 6.9 |
| 2 | 10.06 | Magnetic Island | 19°07.743'S, 146°52.790'E | 16 | 53.2 ± 6.3 | 43 | 65 | 3.8±1.2 | 1.8 | 6.0 |
| 3 | 15.10 | Herald Island | 19°01.683'S, 146°37.753'E | 6 | 48.4 ± 13.2 | 30 | 66 | 3.5±2.7 | 0.6 | 7.9 |
| Offshore |  |  |  |  |  |  |  |  |  |  |
| 5 | 11.07 | Davies Reef | 18°49.793'S, 147°38.046'E | 17 | 63.4 ± 7.4 | 51 | 75 | 5.9±2.2 | 2.7 | 10.9 |
| 4 | 12.07 | John Brewer Reef | 18°38.713'S, 147°02.443'E | 13 | 64.4 ± 8.0 | 51 | 75 | 6.3±2.3 | 3.2 | 9.6 |

**Table S2. Application of analysis workflow (Kroon *et al.* 2018).** The workflow is tailored to quantifying marine microdebris contamination, and applied to (a) 22 surface water tows, and (b) the gastrointestinal tract contents of 60 Lemon damselfish (*Pomacentrus moluccensis*), collected at inshore and offshore reef locations in the central Great Barrier Reef World Heritage Area, Australia, in 2016. The number of items affected by each step of the analysis workflow, and the reason for change in numbers are given. *^b^* percentage based on total items identified during visual separation using stereomicroscopy.

(a)

| **Step** | **Process** | **Number of items processed** | | | | | | | **Reason for change in numbers** |
| --- | --- | --- | --- | --- | --- | --- | --- | --- | --- |
|  |  | **Affected by each step** | | | **Taken to next step** | | | |  |
|  |  | **Total** | **Fibres** | **Particles** | **Total** | **Fibres** | **Particles** | **%*^b^*** |  |
| 1 | Visual identification | - | - | - | 681 | 494 | 187 | - | - |
| 2 | Measurement and photography | 0 | 0 | 0 | 681 | 494 | 187 | 100 | - |
| 3 | Chemical characterisation | 53 | 24 | 29 | 628 | 470 | 158 | 92 | too small for ATR-FTIR |
| 4 | Library interpretation of spectra | 10 | 8 | 2 | 618 | 462 | 156 | 91 | <60% match |
| 5 | Visual inspection of spectra | 14 | 8 | 6 | 604 | 454 | 150 | 89 | 60-<80% with poor visual match |
| 6 | Contamination check | 162 | 99 | 63 | - | - | - | - | ≥90% match to contaminant library |
| 7 | Visual inspection of photographs | 19 | 19 | 0 | 585 | 435 | 150 | 86 | visual match to contaminant |
| 8 | Chemical type assignment | 38 | 24 | 14 | 547 | 411 | 136 | 80 | natural origin could not be excluded |
| **Marine microdebris items** | | | | | **547** | **411** | **136** | **80** |  |

(b)

| **Step** | **Process** | **Number of items** | | | | | | | **Reason for change in numbers** |
| --- | --- | --- | --- | --- | --- | --- | --- | --- | --- |
|  |  | **Affected by each step** | | | **Taken to next step** | | | |  |
|  |  | **Total** | **Fibres** | **Particles** | **Total** | **Fibres** | **Particles** | **%*^b^*** |  |
| 1 | Visual identification | - | - | - | 556 | 546 | 10 | - | - |
| 2 | Measurement and photography | 0 | 0 | 0 | 556 | 546 | 10 | 100 | - |
| 3 | Chemical characterisation | 53 | 51 | 2 | 503 | 495 | 8 | 90 | too small for ATR-FTIR |
| 4 | Library interpretation of spectra | 6 | 6 | 0 | 497 | 489 | 8 | 89 | <60% match |
| 5 | Visual inspection of spectra | 8 | 8 | 0 | 489 | 481 | 8 | 88 | 60-<80% and poor visual match |
| 6 | Contamination check | 61 | 61 | 0 | - | - | - | - | ≥90% match to contaminant library |
| 7 | Visual inspection of photographs | 14 | 14 | 0 | 475 | 467 | 8 | 85 | visual match to contaminant |
| 8 | Chemical type assignment | 20 | 13 | 7 | 455 | 454 | 1 | 82 | natural origin could not be excluded |
| **Marine microdebris items** | | | | | **455** | **454** | **1** | **82** |  |

**Table S3. Customised contaminant libraries.** The libraries for (a) surface water, and (b) fish gastrointestinal tract samples include (i) reference samples of materials from field collection, Research Vessel Cape Ferguson, field processing, and laboratory processing, and (ii) items obtained in procedural blank controls to check for inadvertent airborne contamination.

(a)

| **Source** | **Colour** | **Type** |
| --- | --- | --- |
| RV Cape Ferguson hull paint | blue | particle |
| RV Cape Ferguson hull paint and rust | blue/rusty | particle |
| RV Cape Ferguson rust | rusty | particle |
| RV Cape Ferguson rope - green | green | fibre |
| RV Cape Ferguson rope - white | white | fibre |
| Plankton net - mesh | white | fibre |
| Plankton net - canvas | white | fibre |
| Plankton net - cod end base | transparent | particle |
| 37 µm mesh | transparent | fibre |
| Sample vial - base | transparent | particle |
| Sample vial - lid | yellow | particle |
| Microscope cover | red | fibre |
| Bogorov chamber | transparent | particle |
| White tape - top | white | particle |
| White tape - glue base | white | particle |
| Lab coat | white | fibre |
| Lint free tissue | white | fibre |
| Nitrile glove blue | blue | particle |
| Human skin | brown | particle |
| Rubber band | brown | particle |
| Petridish 1 fibre 1 | black | fibre |
| Petridish 1 fibre 2 | black | fibre |
| Petridish 1 fibre 3 | blue | fibre |
| Petridish 1 fibre 4 | white | fibre |
| Petridish 2 fibre 2 | black | fibre |
| Petridish 2 fibre 3 | white | fibre |
| Petridish 3 fibre 1 | blue | fibre |
| Petridish 3 fibre 2 | blue + white | fibre |
| Petridish 4 fibre 1 | black | fibre |
| Petridish 6 fibre 1 | black | fibre |
| Petridish 7 fibre 1 | black | fibre |
| Petridish 7 fibre 2 | black | fibre |
| Petridish 7 fibre 3 | white | fibre |
| Petridish 8 fibre 1 | blue | fibre |
| Petridish 9 fibre 1 | blue | fibre |
| Petridish 10 fibre 1 | black | fibre |

(b)

| **Source** | **Colour** | **Type** |
| --- | --- | --- |
| Clove oil bottle - container | transparent | particle |
| Fence net | transparent | fibre |
| Fish net - blue | blue | fibre |
| Fish net - green | green | fibre |
| Resealable bag | transparent | particle |
| 37 µm mesh | transparent | fibre |
| Bogorov chamber | transparent | particle |
| Microscope cover | red | fibre |
| White tape - top | white | particle |
| White tape - glue base | white | particle |
| Lab coat | white | fibre |
| Lint free tissue | white | fibre |
| Nitrile glove - green | green | particle |
| Human skin | brown | particle |
| Rubber band | brown | particle |
| Petridish 1 fibre 1 | black | fibre |
| Petridish 1 fibre 2 | black | fibre |
| Petridish 1 fibre 3 | blue | fibre |
| Petridish 1 fibre 4 | black | fibre |
| Petridish 2 fibre 1 | transparent | fibre |
| Petridish 3 fibre 1 | white | fibre |
| Petridish 4 fibre 1 | black | fibre |
| Petridish 4 fibre 2 | red | fibre |
| Petridish 5 fibre 1 | transparent | fibre |
| Petridish 6 fibre 1 | white | fibre |

**Table S4. Spectral matches of potential marine microdebris items with items in customised contaminant libraries.** Spectral matches for potential marine microdebris items separated in (a) surface water, and (b) fish gastrointestinal tract samples were compared to items in their respective customised contaminant library. The number of potential marine microdebris items with spectral matches ≥90% against each individual contaminant item are presented.

(a)

| **Contaminant type** | **Number of sample items** |
| --- | --- |
| RV Cape Ferguson hull paint and rust | 1 |
| RV Cape Ferguson hull paint | 1 |
| RV Cape Ferguson rust | 2 |
| Sample vial - lid | 52 |
| Sample vial - base | 28 |
| Plankton net - canvas | 28 |
| Plankton net - cod end base | 28 |
| White tape | 1 |
| Lab coat | 70 |
| Lint free tissue | 9 |
| Petri dish fibres | 2 |
| **Total** | **222** |

(b)

| **Contaminant type** | **Number of sample items** |
| --- | --- |
| Fish net - blue | 37 |
| Fish net - green | 38 |
| Resealable bag | 3 |
| Lab coat | 41 |
| Lint free tissue | 15 |
| Petridish fibres | 1 |
| **Total** | **135** |

**Table S5. Concentrations and abundance of marine microdebris.** Microdebris (a) concentrations in surface water samples (in m^-3^), and (b) abundance in fish gastrointestinal tract samples (in fish^-1^) are presented for total number of marine microdebris items detected, as well as for total number of fibres and particles, respectively. Collections were conducted near inshore and offshore reef locations in the central region of the Great Barrier Reef World Heritage Area, Australia, 2016. S = synthetic, SS = semi-synthetic, and ND = naturally-derived.

(a)

| **Tow number** | **Location** | **Total number of items (m^-3^)** | | | | **Total number of fibres (m^-3^)** | | | | **Total number of particles (m^-3^)** | | | |
| --- | --- | --- | --- | --- | --- | --- | --- | --- | --- | --- | --- | --- | --- |
|  |  | **S** | **SS** | **ND** | **Total** | **S** | **SS** | **ND** | **Total** | **S** | **SS** | **ND** | **Total** |
| Offshore |  |  |  |  |  |  |  |  |  |  |  |  |  |
| 1 | Kelso reef | 0.06 | 0.02 | 0.03 | 0.11 | 0.03 | 0.02 | 0.03 | 0.08 | 0.03 | 0.00 | 0.00 | 0.03 |
| 2 | Roxburgh reef | 0.07 | 0.07 | 0.02 | 0.15 | 0.04 | 0.07 | 0.02 | 0.12 | 0.03 | 0.00 | 0.00 | 0.03 |
| 3 | Fore and Aft reef | 0.03 | 0.02 | 0.01 | 0.06 | 0.03 | 0.02 | 0.01 | 0.05 | 0.01 | 0.00 | 0.00 | 0.01 |
| 4 | Chicken reef | 0.14 | 0.18 | 0.07 | 0.40 | 0.06 | 0.16 | 0.07 | 0.30 | 0.08 | 0.02 | 0.00 | 0.10 |
| 5 | Grub reef | 0.09 | 0.13 | 0.10 | 0.33 | 0.06 | 0.13 | 0.10 | 0.28 | 0.03 | 0.01 | 0.01 | 0.05 |
| 6 | Little Kelso reef | 0.05 | 0.06 | 0.02 | 0.13 | 0.05 | 0.06 | 0.02 | 0.13 | 0.00 | 0.00 | 0.00 | 0.00 |
| 7 | Rib reef | 0.02 | 0.01 | 0.02 | 0.05 | 0.02 | 0.01 | 0.02 | 0.05 | 0.00 | 0.00 | 0.00 | 0.00 |
| 8 | Centipede reef | 0.10 | 0.12 | 0.04 | 0.25 | 0.04 | 0.12 | 0.04 | 0.19 | 0.07 | 0.00 | 0.00 | 0.07 |
| 9 | Chicken reef | 0.10 | 0.17 | 0.06 | 0.32 | 0.03 | 0.16 | 0.06 | 0.25 | 0.07 | 0.01 | 0.00 | 0.08 |
| 10 | Centipede reef | 0.14 | 0.19 | 0.15 | 0.47 | 0.09 | 0.19 | 0.15 | 0.43 | 0.05 | 0.00 | 0.00 | 0.05 |
| 11 | John Brewer reef | 0.07 | 0.19 | 0.21 | 0.47 | 0.04 | 0.19 | 0.21 | 0.44 | 0.03 | 0.00 | 0.00 | 0.03 |
| Inshore |  |  |  |  |  |  |  |  |  |  |  |  |  |
| 12 | Ross River | 0.10 | 0.01 | 0.01 | 0.12 | 0.01 | 0.01 | 0.01 | 0.03 | 0.09 | 0.00 | 0.00 | 0.09 |
| 13 | #25 | 0.12 | 0.08 | 0.05 | 0.25 | 0.01 | 0.08 | 0.03 | 0.12 | 0.12 | 0.00 | 0.01 | 0.13 |
| 14 | Middle reef | 0.00 | 0.04 | 0.03 | 0.07 | 0.00 | 0.04 | 0.03 | 0.07 | 0.00 | 0.00 | 0.00 | 0.00 |
| 15 | West of Magnetic Island (1) | 0.03 | 0.03 | 0.01 | 0.07 | 0.00 | 0.03 | 0.01 | 0.04 | 0.03 | 0.00 | 0.00 | 0.03 |
| 16 | West of Magnetic Island (2) | 0.00 | 0.04 | 0.00 | 0.04 | 0.00 | 0.04 | 0.00 | 0.04 | 0.00 | 0.00 | 0.00 | 0.00 |
| 17 | West of Magnetic Island (3) | 0.02 | 0.02 | 0.03 | 0.07 | 0.01 | 0.02 | 0.03 | 0.06 | 0.01 | 0.00 | 0.00 | 0.01 |
| 18 | Cockle Bay Reef | 0.04 | 0.03 | 0.10 | 0.17 | 0.02 | 0.03 | 0.10 | 0.14 | 0.02 | 0.00 | 0.00 | 0.02 |
| 19 | Ross River mouth | 0.14 | 0.07 | 0.03 | 0.25 | 0.03 | 0.07 | 0.03 | 0.13 | 0.12 | 0.00 | 0.00 | 0.12 |
| 20 | #39 | 0.03 | 0.03 | 0.02 | 0.08 | 0.01 | 0.02 | 0.02 | 0.05 | 0.02 | 0.01 | 0.00 | 0.03 |
| 21 | Rattlesnake | 0.02 | 0.17 | 0.08 | 0.26 | 0.01 | 0.17 | 0.08 | 0.26 | 0.01 | 0.00 | 0.00 | 0.01 |
| 22 | Palm Island | 0.10 | 0.06 | 0.00 | 0.17 | 0.00 | 0.00 | 0.00 | 0.00 | 0.10 | 0.06 | 0.00 | 0.17 |

(b)

| **Fish number** | **Location** | **Total number of items (fish^-1^)** | | | | **Total number of fibres (fish^-1^)** | | | | **Total number of particles (fish^-1^)** | | | |
| --- | --- | --- | --- | --- | --- | --- | --- | --- | --- | --- | --- | --- | --- |
|  |  | **S** | **SS** | **ND** | **Total** | **S** | **SS** | **ND** | **Total** | **S** | **SS** | **ND** | **Total** |
| Inshore |  |  |  |  |  |  |  |  |  |  |  |  |  |
| 1 | Great Palm Island | 0 | 0 | 4 | 4 | 0 | 0 | 4 | 4 | 0 | 0 | 0 | 0 |
| 2 | Great Palm Island | 2 | 3 | 3 | 8 | 2 | 3 | 3 | 8 | 0 | 0 | 0 | 0 |
| 3 | Great Palm Island | 0 | 0 | 4 | 4 | 0 | 0 | 4 | 4 | 0 | 0 | 0 | 0 |
| 4 | Great Palm Island | 0 | 2 | 3 | 5 | 0 | 2 | 3 | 5 | 0 | 0 | 0 | 0 |
| 5 | Great Palm Island | 0 | 1 | 1 | 2 | 0 | 1 | 1 | 2 | 0 | 0 | 0 | 0 |
| 6 | Great Palm Island | 0 | 4 | 2 | 6 | 0 | 4 | 2 | 6 | 0 | 0 | 0 | 0 |
| 7 | Great Palm Island | 0 | 1 | 4 | 5 | 0 | 1 | 4 | 5 | 0 | 0 | 0 | 0 |
| 8 | Great Palm Island | 0 | 0 | 3 | 3 | 0 | 0 | 3 | 3 | 0 | 0 | 0 | 0 |
| 9 | Magnetic Island | 0 | 6 | 12 | 18 | 0 | 6 | 12 | 18 | 0 | 0 | 0 | 0 |
| 10 | Magnetic Island | 0 | 1 | 3 | 4 | 0 | 1 | 3 | 4 | 0 | 0 | 0 | 0 |
| 11 | Magnetic Island | 1 | 0 | 1 | 2 | 1 | 0 | 1 | 2 | 0 | 0 | 0 | 0 |
| 12 | Magnetic Island | 0 | 5 | 2 | 7 | 0 | 5 | 2 | 7 | 0 | 0 | 0 | 0 |
| 13 | Magnetic Island | 0 | 1 | 3 | 4 | 0 | 1 | 3 | 4 | 0 | 0 | 0 | 0 |
| 14 | Magnetic Island | 0 | 2 | 3 | 5 | 0 | 2 | 3 | 5 | 0 | 0 | 0 | 0 |
| 15 | Magnetic Island | 1 | 3 | 1 | 5 | 1 | 3 | 1 | 5 | 0 | 0 | 0 | 0 |
| 16 | Magnetic Island | 0 | 9 | 5 | 14 | 0 | 9 | 5 | 14 | 0 | 0 | 0 | 0 |
| 17 | Magnetic Island | 0 | 1 | 0 | 1 | 0 | 1 | 0 | 1 | 0 | 0 | 0 | 0 |
| 18 | Magnetic Island | 3 | 1 | 3 | 7 | 3 | 1 | 3 | 7 | 0 | 0 | 0 | 0 |
| 19 | Magnetic Island | 0 | 0 | 0 | 0 | 0 | 0 | 0 | 0 | 0 | 0 | 0 | 0 |
| 20 | Magnetic Island | 1 | 1 | 3 | 5 | 1 | 1 | 3 | 5 | 0 | 0 | 0 | 0 |
| 21 | Magnetic Island | 0 | 1 | 2 | 3 | 0 | 1 | 2 | 3 | 0 | 0 | 0 | 0 |
| 22 | Magnetic Island | 1 | 0 | 0 | 1 | 1 | 0 | 0 | 1 | 0 | 0 | 0 | 0 |
| 23 | Magnetic Island | 0 | 1 | 2 | 3 | 0 | 1 | 2 | 3 | 0 | 0 | 0 | 0 |
| 24 | Magnetic Island | 0 | 1 | 2 | 3 | 0 | 1 | 2 | 3 | 0 | 0 | 0 | 0 |
| 25 | Herald Island | 1 | 1 | 2 | 4 | 1 | 1 | 2 | 4 | 0 | 0 | 0 | 0 |
| 26 | Herald Island | 0 | 3 | 1 | 4 | 0 | 3 | 1 | 4 | 0 | 0 | 0 | 0 |
| 27 | Herald Island | 1 | 4 | 2 | 7 | 1 | 4 | 2 | 7 | 0 | 0 | 0 | 0 |
| 28 | Herald Island | 0 | 2 | 7 | 9 | 0 | 2 | 7 | 9 | 0 | 0 | 0 | 0 |
| 29 | Herald Island | 1 | 2 | 6 | 9 | 1 | 2 | 6 | 9 | 0 | 0 | 0 | 0 |
| 30 | Herald Island | 1 | 0 | 7 | 8 | 1 | 0 | 7 | 8 | 0 | 0 | 0 | 0 |
| Offshore |  |  |  |  |  |  |  |  |  |  |  |  |  |
| 31 | Davies Reef | 1 | 20 | 14 | 35 | 1 | 20 | 14 | 35 | 0 | 0 | 0 | 0 |
| 32 | Davies Reef | 0 | 2 | 5 | 7 | 0 | 2 | 5 | 7 | 0 | 0 | 0 | 0 |
| 33 | Davies Reef | 0 | 0 | 1 | 1 | 0 | 0 | 1 | 1 | 0 | 0 | 0 | 0 |
| 34 | Davies Reef | 2 | 5 | 9 | 16 | 2 | 5 | 9 | 16 | 0 | 0 | 0 | 0 |
| 35 | Davies Reef | 1 | 0 | 1 | 2 | 1 | 0 | 1 | 2 | 0 | 0 | 0 | 0 |
| 36 | Davies Reef | 0 | 2 | 1 | 3 | 0 | 2 | 1 | 3 | 0 | 0 | 0 | 0 |
| 37 | Davies Reef | 0 | 2 | 0 | 2 | 0 | 2 | 0 | 2 | 0 | 0 | 0 | 0 |
| 38 | Davies Reef | 0 | 2 | 2 | 4 | 0 | 2 | 2 | 4 | 0 | 0 | 0 | 0 |
| 39 | Davies Reef | 0 | 2 | 5 | 7 | 0 | 2 | 5 | 7 | 0 | 0 | 0 | 0 |
| 40 | Davies Reef | 0 | 1 | 2 | 3 | 0 | 1 | 2 | 3 | 0 | 0 | 0 | 0 |
| 41 | Davies Reef | 0 | 1 | 4 | 5 | 0 | 0 | 4 | 4 | 0 | 1 | 0 | 1 |
| 42 | Davies Reef | 0 | 0 | 2 | 2 | 0 | 0 | 2 | 2 | 0 | 0 | 0 | 0 |
| 43 | Davies Reef | 0 | 0 | 2 | 2 | 0 | 0 | 2 | 2 | 0 | 0 | 0 | 0 |
| 44 | Davies Reef | 0 | 2 | 3 | 5 | 0 | 2 | 3 | 5 | 0 | 0 | 0 | 0 |
| 45 | Davies Reef | 0 | 0 | 1 | 1 | 0 | 0 | 1 | 1 | 0 | 0 | 0 | 0 |
| 46 | Davies Reef | 0 | 0 | 0 | 0 | 0 | 0 | 0 | 0 | 0 | 0 | 0 | 0 |
| 47 | Davies Reef | 0 | 4 | 0 | 4 | 0 | 4 | 0 | 4 | 0 | 0 | 0 | 0 |
| 48 | John Brewer Reef | 0 | 2 | 2 | 4 | 0 | 2 | 2 | 4 | 0 | 0 | 0 | 0 |
| 49 | John Brewer Reef | 0 | 1 | 1 | 2 | 0 | 1 | 1 | 2 | 0 | 0 | 0 | 0 |
| 50 | John Brewer Reef | 2 | 8 | 5 | 15 | 2 | 8 | 5 | 15 | 0 | 0 | 0 | 0 |
| 51 | John Brewer Reef | 0 | 2 | 9 | 11 | 0 | 2 | 9 | 11 | 0 | 0 | 0 | 0 |
| 52 | John Brewer Reef | 0 | 0 | 2 | 2 | 0 | 0 | 2 | 2 | 0 | 0 | 0 | 0 |
| 53 | John Brewer Reef | 0 | 4 | 1 | 5 | 0 | 4 | 1 | 5 | 0 | 0 | 0 | 0 |
| 54 | John Brewer Reef | 35 | 28 | 68 | 131 | 35 | 28 | 68 | 131 | 0 | 0 | 0 | 0 |
| 55 | John Brewer Reef | 0 | 2 | 3 | 5 | 0 | 2 | 3 | 5 | 0 | 0 | 0 | 0 |
| 56 | John Brewer Reef | 0 | 0 | 0 | 0 | 0 | 0 | 0 | 0 | 0 | 0 | 0 | 0 |
| 57 | John Brewer Reef | 0 | 2 | 2 | 4 | 0 | 2 | 2 | 4 | 0 | 0 | 0 | 0 |
| 58 | John Brewer Reef | 0 | 4 | 3 | 7 | 0 | 4 | 3 | 7 | 0 | 0 | 0 | 0 |
| 59 | John Brewer Reef | 0 | 1 | 7 | 8 | 0 | 1 | 7 | 8 | 0 | 0 | 0 | 0 |
| 60 | John Brewer Reef | 0 | 1 | 1 | 2 | 0 | 1 | 1 | 2 | 0 | 0 | 0 | 0 |

# Figures


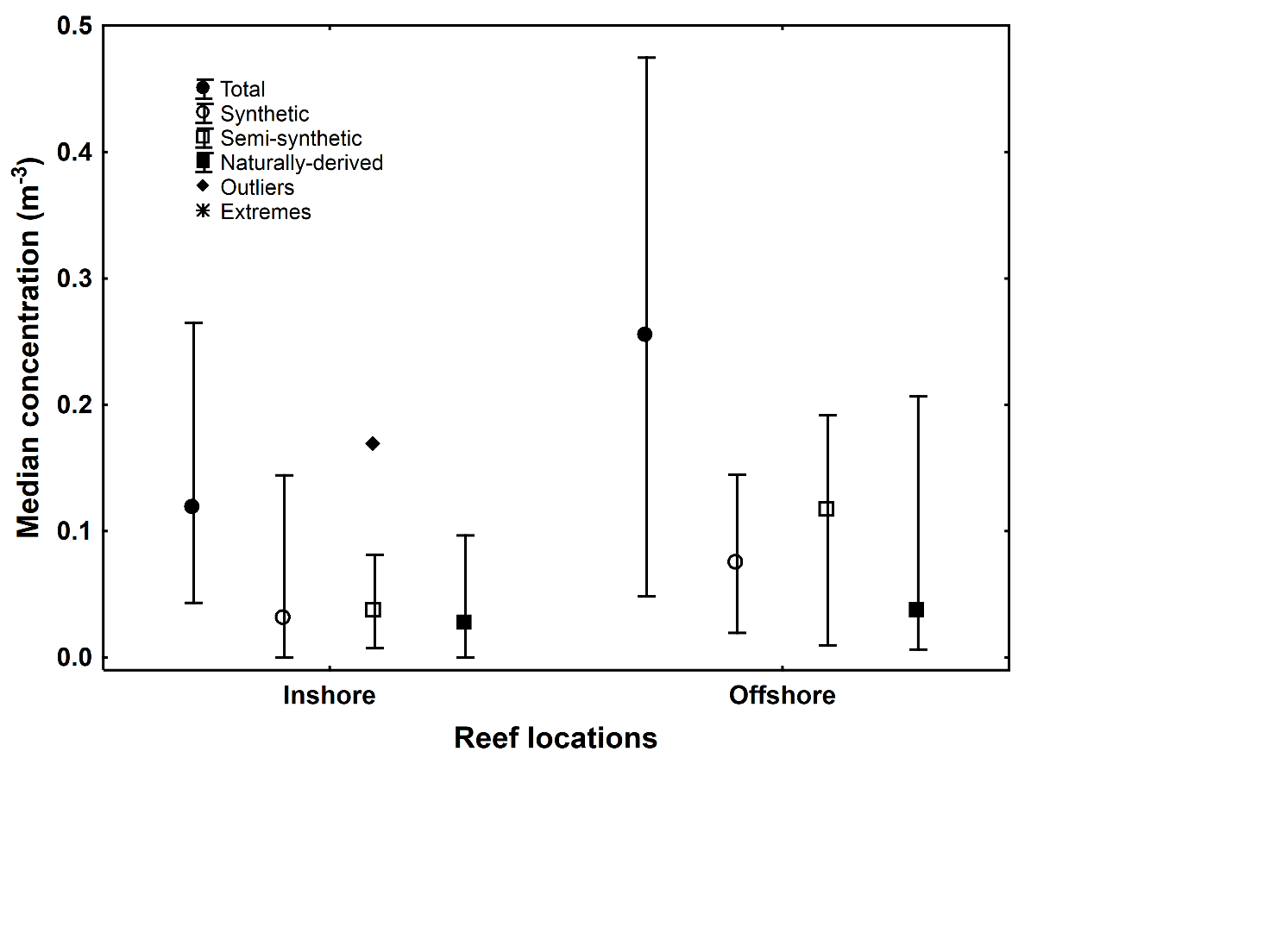


**Figure S1. Concentrations of marine microdebris in surface water tows.** Median concentration of total marine microdebris (fibres and particles) in 22 surface water tows conducted near inshore (n=11) and offshore (n=11) reef locations in the central region of the Great Barrier Reef World Heritage Area, Australia, 2016. Microdebris is classified as total, synthetic, semi-synthetic, and naturally-derived items. Whiskers show non-outlier range.


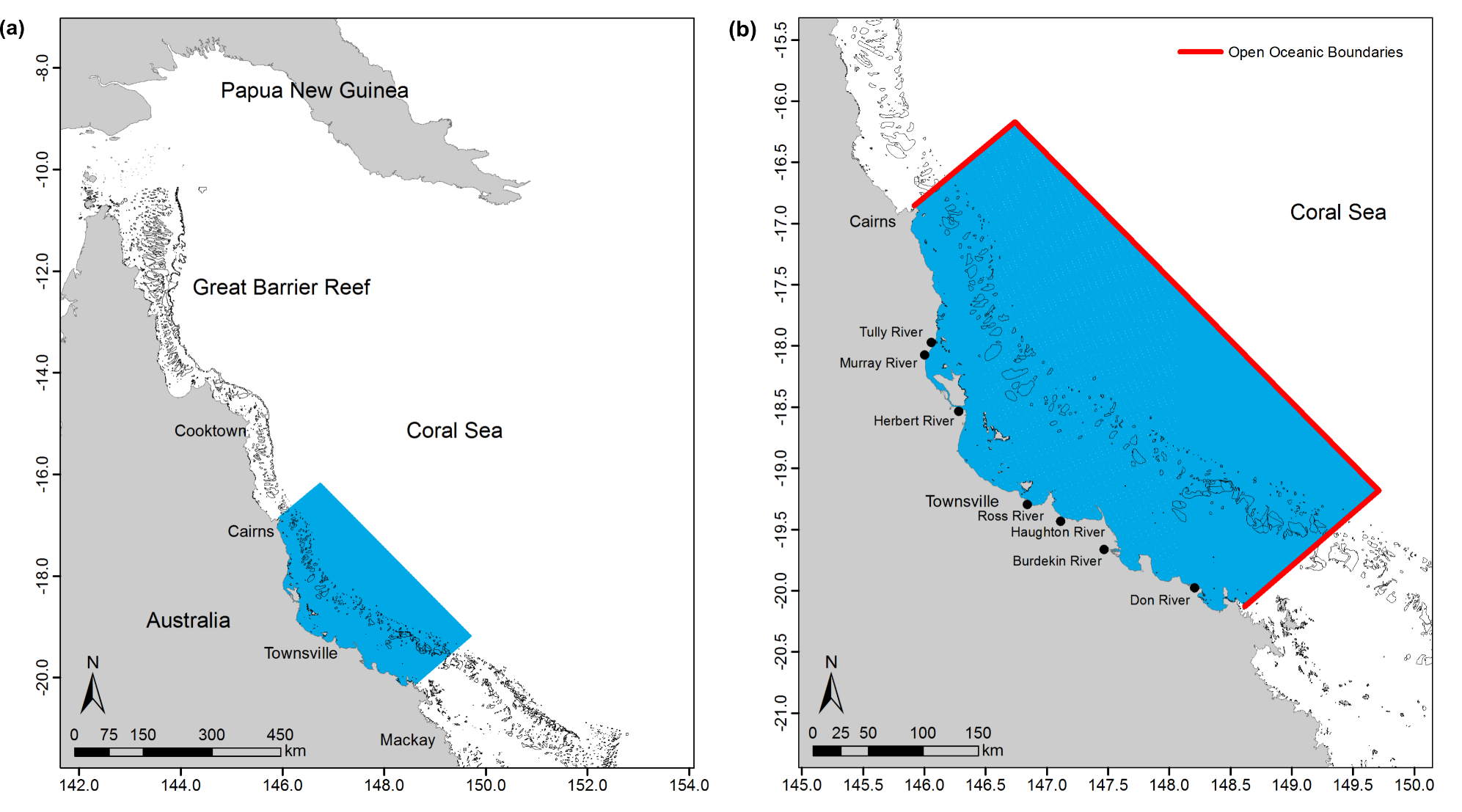


**Figure S2**. **The modelling domain (blue area) for the two numerical simulations**. The domain encompasses the study area in the central Great Barrier Reef, Australia, where collection of surface water samples was conducted at both inshore and offshore reef locations. The modelling domain is presented in the context of (**a**) northeast Australia showing the main coastal cities, and (**b**) the central Great Barrier Reef showing the main coastal rivers used in the simulations (Don, Burdekin, Haughton, Ross, Herbert, Murray and Tully), and the open oceanic boundaries (red). Coral reefs are presented as black.


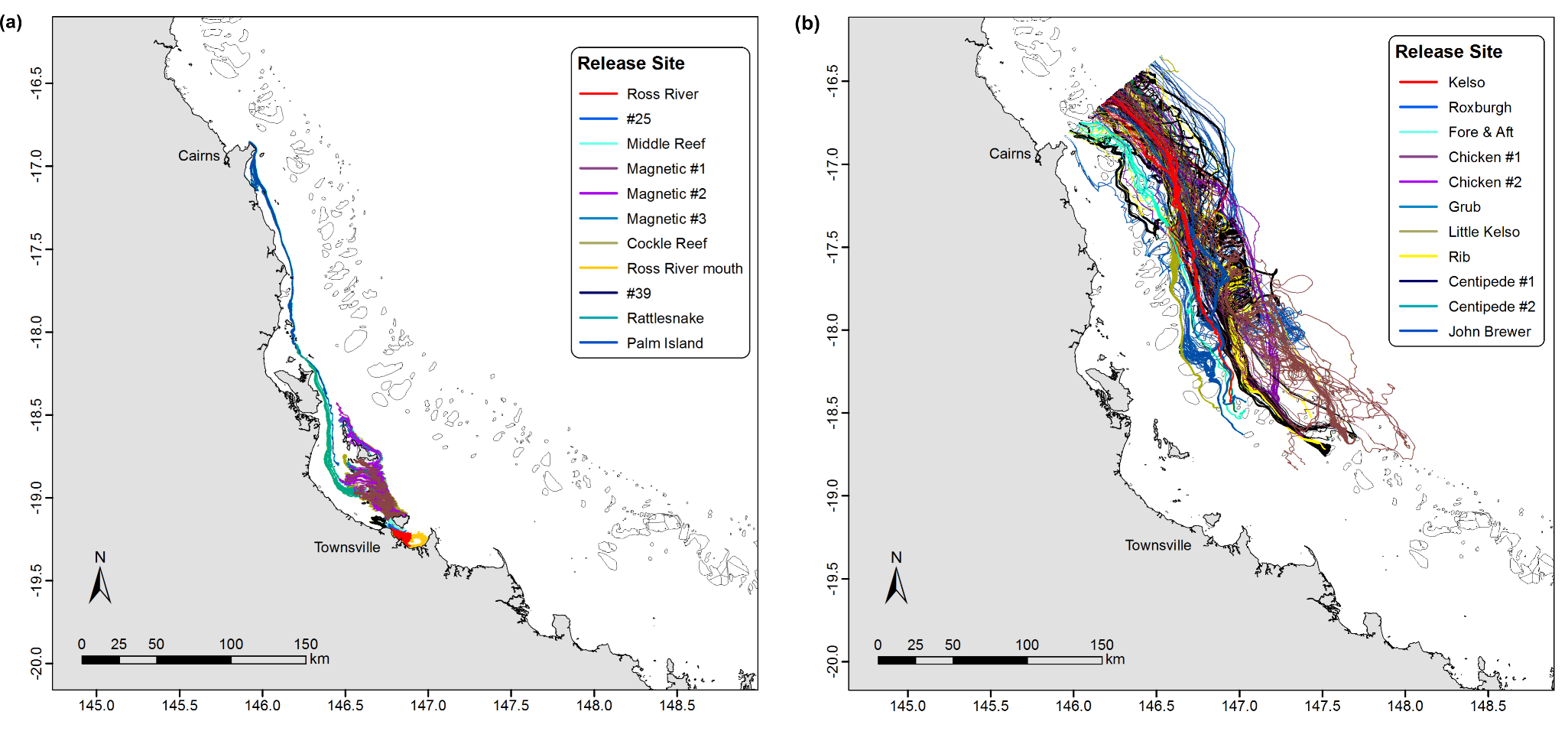
 **Figure S3**. **Virtual drogues trajectories released at the 22 individual sampling locations**. Thirty-day trajectories of virtual drogues released at 22 individual sampling locations in the (a) inshore (n=11), and (b) offshore (n=11) regions of the central Great Barrier Reef. Only every second release (i.e. every two minutes) is shown to avoid overexposure of and better visually define trajectories. The cessation of the trajectories (observed as a straight line) indicates that the drogues leave the numerical domain.

# References

1. Deltares. Delft3D-FLOW. Simulation of multi-dimensional hydrodynamic flows and transport phenomena, including sediments. Version: 3.15, Revision 45038, 644 (User Manual, Deltares, Delft, The Netherlands, 2016).

2. Beaman, R. J. Project 3D-GBR: A high-resolution depth model for the Great Barrier Reef and Coral Sea. Project 2.5i.1a, 13 (Final Report, Marine and Tropical Sciences Research Facility, Cairns, Australia, 2010).

3. Herzfeld, M. An alternative coordinate system for solving finite difference ocean models. *Ocean Model.* **14**, 174–196 (2006).

4. Herzfeld, M.*, et al.* eReefs marine modelling, 497 (Final Report, CSIRO, Hobart, Australia, 2016).

5. Schiller, A., Herzfeld, M., Brinkman, R. & Stuart, G. Monitoring, predicting, and managing one of the seven natural wonders of the world. *Bull. Am. Meteorol. Soc.* **95**, 23–30 (2014).
